# Supplementary material for: Single-Stranded Phosphorothioated Regions Enhance Cellular Uptake of Cholesterol-Conjugated siRNA but Not Silencing Efficacy
Source: Mol Ther Nucleic Acids. 2020 Jul 25;21:991–1005. doi: 10.1016/j.omtn.2020.07.029 (PMC7452107; doi:10.1016/j.omtn.2020.07.029)
Supplement: Document S1. Figures S1–S3 and Table S1 [file mmc1.pdf]

OMTN, Volume *21*

Supplemental Information

Single-Stranded Phosphorothioated Regions Enhance Cellular Uptake of Cholesterol-Conjugated siRNA but Not Silencing Efficacy

Socheata Ly, Dimas Echeverria, Jacquelyn Sousa, and Anastasia Khvorova

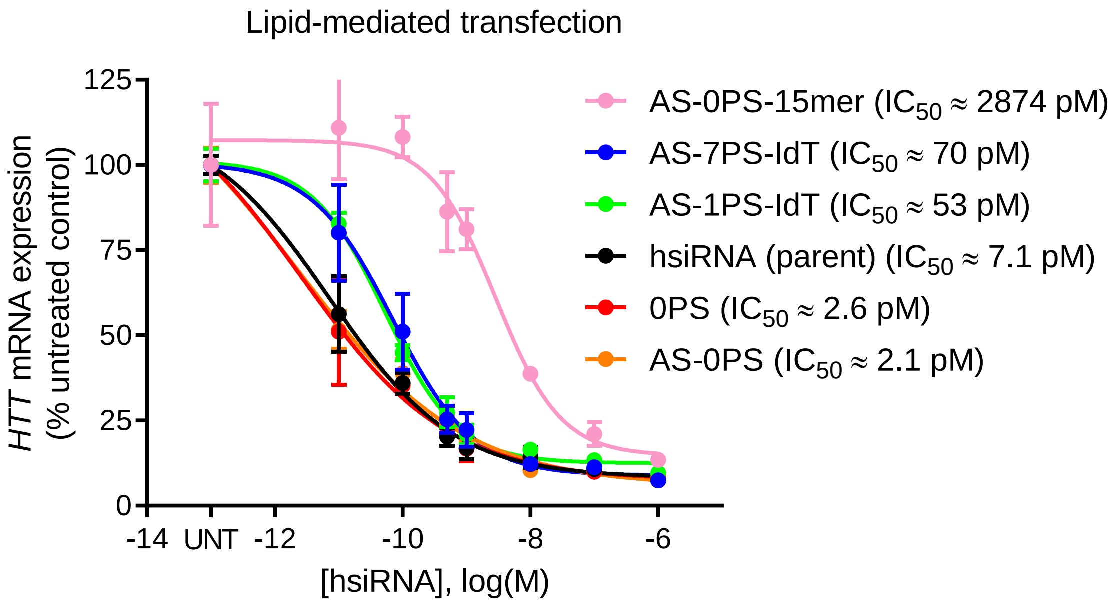

Supplementary Figure 1. PS modifications used in this study are tolerated by RISC, but not a shorter 15-15mer or oligos containing 3′ idT. Seven-point dose response performed in HeLa cells transfected with RNAiMAX. *HTT* mRNA levels were quantified by QuantiGene Assay (Thermo Fisher) after 72 h treatment with hsiRNA. Representative data shown as mean ± SD (n = 3 technical replicates) from at least two independent experiments. UNT = untreated.

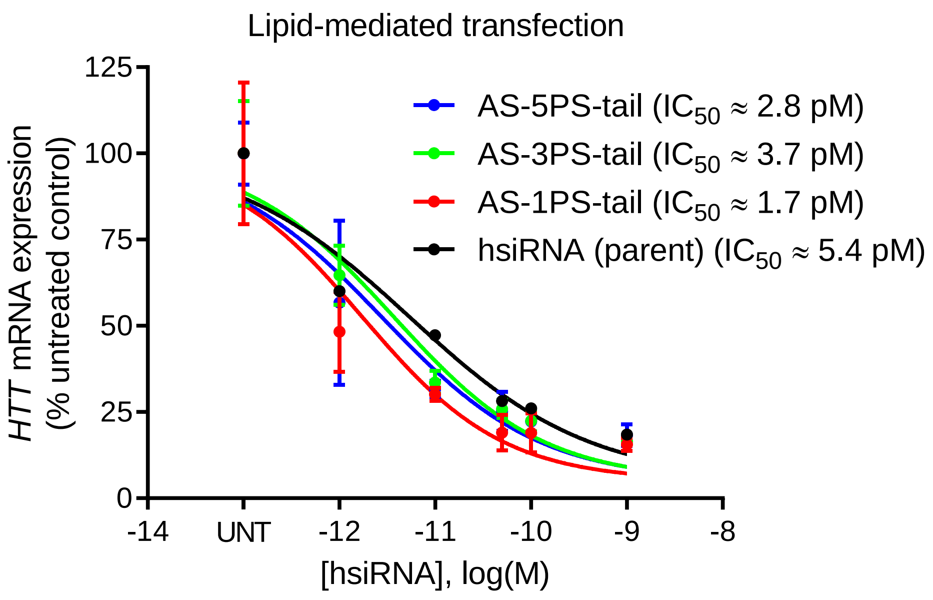

Supplementary Figure 2. Oligos containing reduced PS tails load into RISC as well as the parent compound. Seven-point dose response performed in HeLa cells transfected with RNAiMAX. *HTT* mRNA levels were quantified by QuantiGene Assay (Thermo Fisher) after 72 h treatment with hsiRNA. Representative data shown as mean ± SD (n = 3 technical replicates) from at least two independent experiments. UNT = untreated.

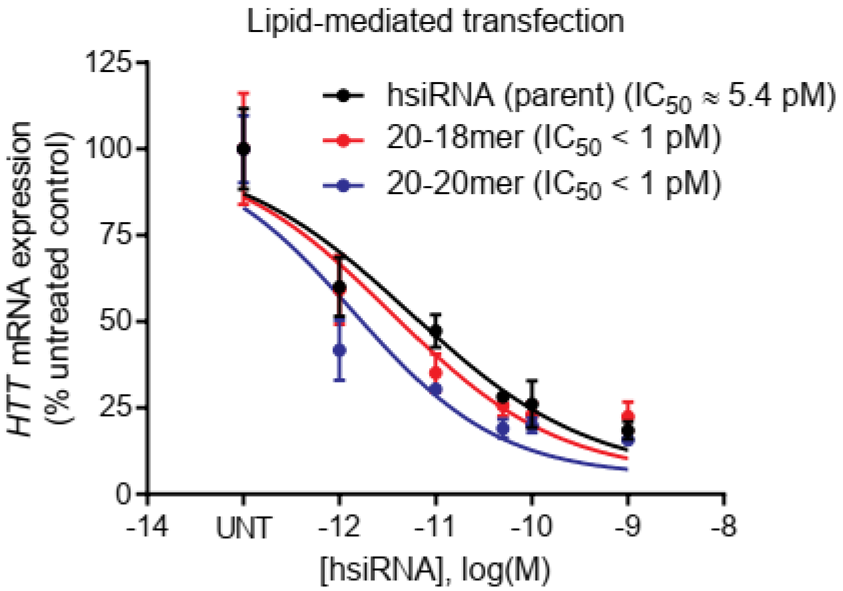

Supplementary Figure 3. 20-20mer and 20-18mer oligos can efficiently silence mRNA via the RNAi pathway. Seven-point dose response performed in HeLa cells transfected with RNAiMAX. *HTT* mRNA levels were quantified by QuantiGene Assay (Thermo Fisher) after 72 h treatment with hsiRNA. Representative data shown as mean ± SD (n = 3 technical replicates) from at least two independent experiments. UNT = untreated.

oligo	strand	sequence (5′ to 3′)
HTT	antisense	P(mU)#(fU)#(mA)(fA)(mU)(fC)(mU)(fC)(mU)(fU)(mU)(fA)(mC)#(fU)#(mG)#(fA)#(mU)#(fA)#(mU)#(fA)
HTT	sense	CyMN3-(fC)#(mA)#(fG)(mU)(fA)(mA)(fA)(mG)(fA)(mG)(fA)(mU)(fU)#(mA)#(fA)-TegChol
sFLT	antisense	P(mU)#(fA)#(mA)(fA)(mU)(fU)(mU)(fG)(mG)(fA)(mG)(fA)(mU)#(fC)#(mC)#(fG)#(mA)#(fG)#(mA)#(fG)
sFLT	sense	CyMN3-(fG)#(mG)#(fA)(mU)(fC)(mU)(fC)(mC)(fA)(mA)(fA)(mU)(fU)#(mU)#(fA)-TegChol
AS-1PS	antisense	P(mU)#(fU)#(mA)(fA)(mU)(fC)(mU)(fC)(mU)(fU)(mU)(fA)(mC)(fU)(mG)(fA)(mU)(fA)(mU)#(fA)
AS-1PS	sense	CyMN3-(fC)#(mA)#(fG)(mU)(fA)(mA)(fA)(mG)(fA)(mG)(fA)(mU)(fU)#(mA)#(fA)-TegChol
AS-3PS	antisense	P(mU)#(fU)#(mA)(fA)(mU)(fC)(mU)(fC)(mU)(fU)(mU)(fA)(mC)(fU)(mG)(fA)(mU)#(fA)#(mU)#(fA)
AS-3PS	sense	CyMN3-(fC)#(mA)#(fG)(mU)(fA)(mA)(fA)(mG)(fA)(mG)(fA)(mU)(fU)#(mA)#(fA)-TegChol
AS-5PS	antisense	P(mU)#(fU)#(mA)(fA)(mU)(fC)(mU)(fC)(mU)(fU)(mU)(fA)(mC)(fU)(mG)#(fA)#(mU)#(fA)#(mU)#(fA)
AS-5PS	sense	CyMN3-(fC)#(mA)#(fG)(mU)(fA)(mA)(fA)(mG)(fA)(mG)(fA)(mU)(fU)#(mA)#(fA)-TegChol
20-20mer	antisense	P(mU)#(fA)#(mA)(fA)(mU)(fU)(mU)(fG)(mG)(fA)(mG)(fA)(mU)#(fC)#(mC)#(fG)#(mA)#(fG)#(mA)#(fG)
20-20mer	sense	CyMN3-(mU)#(fA)#(mU)(fA)(mU)(fC)(mA)(fG)(mU)(fA)(mA)(fA)(mG)(fA)(mG)(fA)(mU)(fU)#(mA)#(fA)-TegChol
20-18mer	antisense	P(mU)#(fA)#(mA)(fA)(mU)(fU)(mU)(fG)(mG)(fA)(mG)(fA)(mU)#(fC)#(mC)#(fG)#(mA)#(fG)#(mA)#(fG)
20-18mer	sense	CyMN3-(mU)#(fA)#(mU)(fC)(mA)(fG)(mU)(fA)(mA)(fA)(mG)(fA)(mG)(fA)(mU)(fU)#(mA)#(fA)-TegChol
HTT15/sFLT5	antisense	P(mU)#(fU)#(mA)(fA)(mU)(fC)(mU)(fC)(mU)(fU)(mU)(fA)(mC)#(fU)#(mG)#(fG)#(mA)#(fG)#(mA)#(fG)
HTT15/sFLT5	sense	CyMN3-(fC)#(mA)#(fG)(mU)(fA)(mA)(fA)(mG)(fA)(mG)(fA)(mU)(fU)#(mA)#(fA)-TegChol
sFLT15/HTT5	antisense	P(mU)#(fA)#(mA)(fA)(mU)(fU)(mU)(fG)(mG)(fA)(mG)(fA)(mU)#(fC)#(mC)#(fA)#(mU)#(fA)#(mU)#(fA)
sFLT15/HTT5	sense	CyMN3-(fG)#(mG)#(fA)(mU)(fC)(mU)(fC)(mC)(fA)(mA)(fA)(mU)(fU)#(mU)#(fA)-TegChol
0PS	antisense	P(mU)(fU)(mA)(fA)(mU)(fC)(mU)(fC)(mU)(fU)(mU)(fA)(mC)(fU)(mG)(fA)(mU)(fA)(mU)(fA)
0PS	sense	CyMN3-(fC)(mA)(fG)(mU)(fA)(mA)(fA)(mG)(fA)(mG)(fA)(mU)(fU)(mA)(fA)-TegChol
AS-0PS	antisense	P(mU)(fU)(mA)(fA)(mU)(fC)(mU)(fC)(mU)(fU)(mU)(fA)(mC)(fU)(mG)(fA)(mU)(fA)(mU)(fA)
AS-0PS	sense	CyMN3-(fC)#(mA)#(fG)(mU)(fA)(mA)(fA)(mG)(fA)(mG)(fA)(mU)(fU)#(mA)#(fA)-TegChol
AS-7PS-idT	antisense	P(mU)#(fU)#(mA)(fA)(mU)(fC)(mU)(fC)(mU)(fU)(mU)(fA)(mC)#(fU)#(mG)#(fA)#(mU)#(fA)#(mU)#(reverse dT)
AS-7PS-idT	sense	CyMN3-(fC)#(mA)#(fG)(mU)(fA)(mA)(fA)(mG)(fA)(mG)(fA)(mU)(fU)#(mA)#(fA)-TegChol
AS-1PS-idT	antisense	P(mU)(fU)(mA)(fA)(mU)(fC)(mU)(fC)(mU)(fU)(mU)(fA)(mC)(fU)(mG)(fA)(mU)(fA)(mU)#(reverse dT)
AS-1PS-idT	sense	CyMN3-(fC)#(mA)#(fG)(mU)(fA)(mA)(fA)(mG)(fA)(mG)(fA)(mU)(fU)#(mA)#(fA)-TegChol
AS-0PS-15mer	antisense	P(mU)(fU)(mA)(fA)(mU)(fC)(mU)(fC)(mU)(fU)(mU)(fA)(mC)(fU)(mG)
AS-0PS-15mer	sense	CyMN3-(fC)#(mA)#(fG)(mU)(fA)(mA)(fA)(mG)(fA)(mG)(fA)(mU)(fU)#(mA)#(fA)-TegChol
No cholesterol	antisense	P(mU)#(fU)#(mA)(fA)(mU)(fC)(mU)(fC)(mU)(fU)(mU)(fA)(mC)#(fU)#(mG)#(fA)#(mU)#(fA)#(mU)#(fA)
No cholesterol	sense	CyMN3-(fC)#(mA)#(fG)(mU)(fA)(mA)(fA)(mG)(fA)(mG)(fA)(mU)(fU)#(mA)#(fA)
**KEY**		
P	5′ phosphate	
mN	2′-O-methyl	
fN	2′-flouro	
#	phosphorothioate	
CyMN3	Cy3	
TegChol	tetra ethylene glycol cholesterol	
reverse dT	3′ to 3′ reverse dT	

Supplementary Table 1. List of oligonucleotides used in this study. See Materials and Methods for information on how the oligonucleotides were synthesized. Key: P, 5′ phosphate; mN, 2′-O-methyl; fN, 2′-flouro; #, phosphorothioate, CyMN3, Cy3; TegChol, tetra ethylene glycol cholesterol; reverse dT, 3′ to 3′ reverse dT.
