# Supplementary material for: Single-Stranded Phosphorothioated Regions Enhance Cellular Uptake of Cholesterol-Conjugated siRNA but Not Silencing Efficacy
Source: Mol Ther Nucleic Acids. 2020 Jul 25;21:991–1005. doi: 10.1016/j.omtn.2020.07.029 (PMC7452107; doi:10.1016/j.omtn.2020.07.029)
Supplement: Document S2. Article plus Supplemental Information [file mmc2.pdf]

Molecular Therapy
Nucleic Acids
Original Article

Single-Stranded Phosphorothioated Regions Enhance Cellular Uptake of Cholesterol-Conjugated siRNA but Not Silencing Efficacy

Socheata Ly,[1] Dimas Echeverria,[1] Jacquelyn Sousa,[1] and Anastasia Khvorova[1]

[1]RNA Therapeutics Institute, University of Massachusetts Medical School, Worcester, MA 01605, USA

Small interfering RNAs (siRNAs) have potential to silence virtually any disease-causing gene but require chemical modifications for delivery to the tissue and cell of interest. Previously, we demonstrated that asymmetric, phosphorothioate (PS)-modified, chemically stabilized, cholesterol-conjugated siRNAs, called hsiRNAs, support rapid cellular uptake and efficient mRNA silencing both in cultured cells and *in vivo*. Here, we systematically evaluated the impact of number, structure, and sequence context of PS-modified backbones on cellular uptake and RNAi-mediated silencing efficacy. We find that PS enhances cellular internalization in a sequence-dependent manner but only when present in a single-stranded but not double-stranded region. Furthermore, the observed increase in cellular internalization did not correlate with functional silencing improvement, indicating that PS-mediated uptake may drive compounds to non-productive sinks. Thus, the primary contributing factor of PS modifications to functional efficacy is likely stabilization rather than enhanced cellular uptake. A better understanding of the relative impact of different chemistries on productive versus non-productive uptake will assist in improved design of therapeutic RNAs.

INTRODUCTION

Small interfering RNA (siRNA)-based drugs have therapeutic potential to specifically target and silence any disease-causing gene.[1–3] However, naked siRNAs are prone to degradation by nucleases as well as too large and too hydrophilic to be internalized into cells on their own. To overcome these physiological barriers, extensive chemical modifications are necessary to provide protection from nucleases and allow the oligonucleotides to enter the cell. Currently, conjugate-mediated delivery is being realized as the clinically dominant delivery paradigm, with a single administration supporting up to 6- to 12-month silencing efficacy in liver, brain, and other tissues evaluated in different models ranging from rodents to humans.[4,5] Sustained therapeutic efficacy relies on conjugate-mediated delivery in conjunction with extensive chemical stabilization. A broad range of conjugates can be used to enhance cellular internalization, including N-acetylgalactosamine (GalNAc),[6] cholesterol,[7,8] and docosahexaenoic acid (DHA),[9] which allow the compounds to be internalized through a subset of the endocytic pathway, accumulating to a significant extent in endosomes and lysosomes.[10–12] Endosomal entrapment is believed to create an intracellular depot of oligonucleotides, and subsequent slow cytoplasmic release results in continuous loading of the endogenous RNAi enzymatic machinery, the RNA-induced silencing complex (RISC), translating into multi-month efficacy. This model relies on the stability of the oligonucleotides inside the highly aggressive biological environment, such as lysosomes, and thus requires extensive chemical stabilization.

A combination of sugar and backbone modification chemistries is necessary to support sustained silencing. Phosphorothioate (PS) is the most commonly used backbone modification, since it does not dramatically change the overall RNA structure and thus is highly compatible with multiple enzymatic interactions inside the cell. Indeed, PS modifications are principally responsible for tissue distribution and cellular internalization of antisense oligonucleotides (ASOs), another class of therapeutic RNAs.[13] There are multiple studies looking at the mechanism of PS contribution on cellular internalization, but it is likely due to enhanced hydrophobicity and thus multiple interactions with serum proteins, cellular receptors, and trafficking proteins.[14–16] In siRNAs, introduction of PS modification enhances terminal stability and is used in all clinically advanced modification patterns and believed to contribute to the efficiency of cellular uptake.[13,17,18]

We have previously extensively characterized the behavior in cultured cells and *in vivo* of a cholesterol-conjugated, asymmetric, fully chemically stabilized siRNA with an alternating 2′-fluoro/2′-O-methyl pattern along with PS on all four termini of the duplex, called hsiRNA. hsiRNA contains a single-stranded, 5-nt overhang at the 3′ end of the antisense strand that is fully PS-modified (PS tail), which is believed to enhance cellular uptake by a mechanism similar to ASO cellular internalization.[11,19] When compared *in vivo*, the presence of the fully phosphorothioated tail results in significantly enhanced tissue accumulation of modified siRNAs even with different conjugates.[20] On the other hand, fully PS-modified oligonucleotides can elicit strong

Received 4 June 2020; accepted 22 July 2020;
https://doi.org/10.1016/j.omtn.2020.07.029.

Correspondence: Anastasia Khvorova, RNA Therapeutics Institute, University of Massachusetts Medical School, Worcester, MA 01605, USA.
E-mail: anastasia.khvorova@umassmed.edu

This is an open access article under the CC BY license (http://creativecommons.org/licenses/by/4.0/).

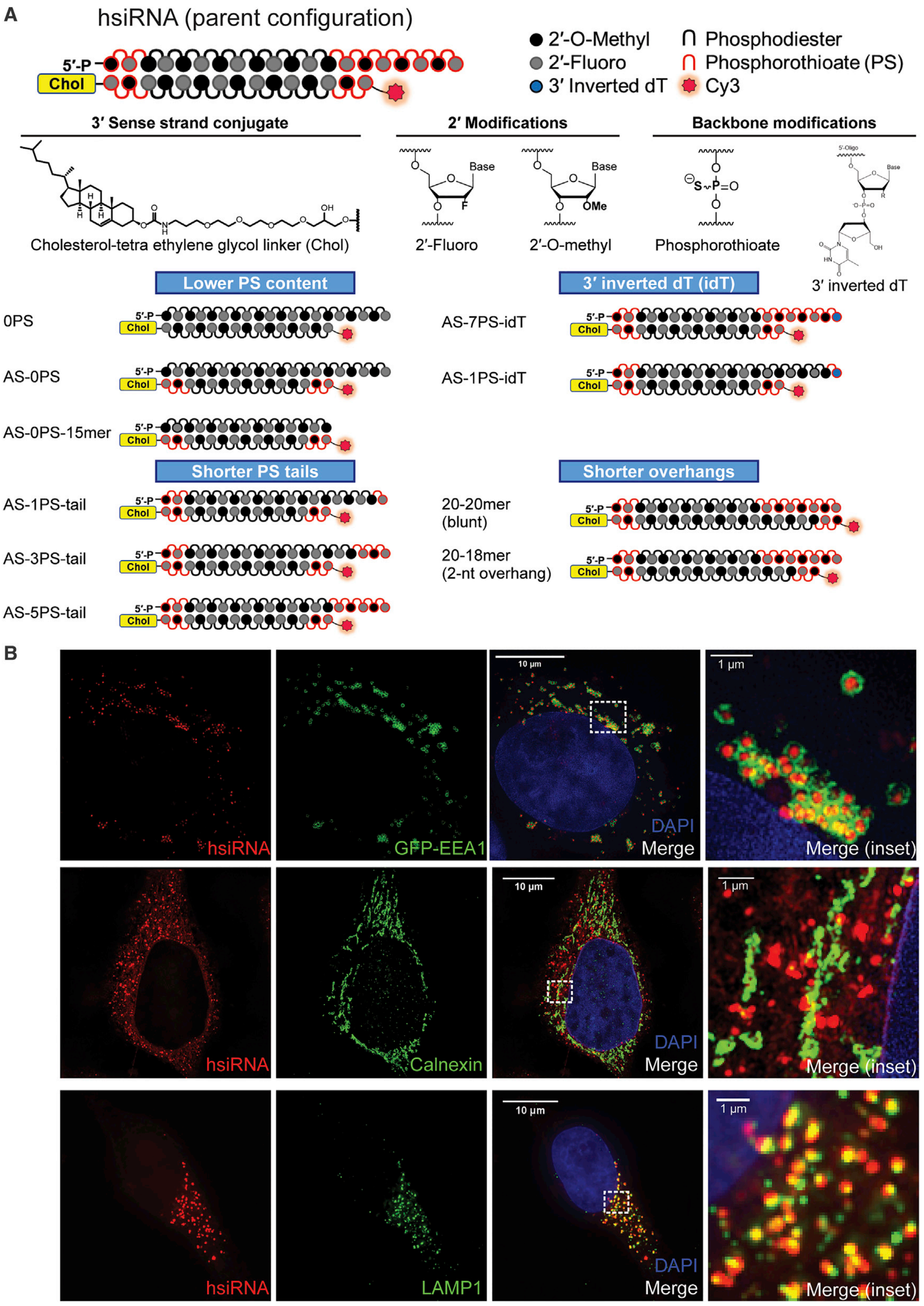

(legend on next page)

toxicity,[15,21] whereas reduction in PS content has been shown to be a successful strategy for the development of compounds with a better safety profile.[22,23]

To this end, we sought to systematically evaluate the impact of the structure (single-stranded versus double-stranded) and extent of PS modifications on the efficiency of hydrophobic siRNA cellular internalization and RISC-mediated silencing. We demonstrate that reduction of PS content results in a significant decrease of the cellular uptake but is not directly correlated to loss of silencing efficacy.

RESULTS

Structures and Chemical Modification Patterns of Self-Delivering siRNA Compounds Used in the Study

In order to evaluate the extent to which the structural context of PS modifications affects the intracellular trafficking of hydrophobically modified siRNAs, we synthesized a panel of variants of a well-studied hydrophobic siRNA configuration called hsiRNA (parent).[19] hsiRNAs are asymmetric (20-nt antisense strand, 15-nt sense strand) siRNAs where all sugars are modified with an alternating 2′-fluoro (F)/2′-O-methyl (OMe) pattern, terminal backbones are protected with PS, and the sense strand is modified with cholesterol. The parent configuration contains seven PS modifications on the antisense 3′ end, generating a 5-nt-long fully phosphorothioated tail. The panel consisted of compounds lacking PS completely, only in the antisense strand, as well as asymmetric configurations with different numbers of PS modifications on the antisense 3′ end (Figure 1). In addition, we changed the length of the sense strand (15-, 18-, and 20-mer) to evaluate the degree of importance of the single-stranded nature of the PS tail for cellular uptake.

hsiRNAs are efficiently internalized by cells through simple addition to the media. Uptake is mostly driven by the early endosome antigen 1 (EEA1)-associated endocytic pathway in COS-7 or HeLa cells.[11] The oligonucleotides are labeled with Cy3, which allows evaluation of intracellular distribution. Consistent with previously reported data, we observed significant entrapment of internalized siRNAs in EEA1-enriched endosomes in both COS-7 and HeLa cells (Figure 1B).[11] Interestingly, there is practically no visual co-localization with calnexin (rough endoplasmic reticulum [ER] marker, putative RISC-nucleation point)[24] at 24 h, indicating that the majority of visually detectable oligonucleotides are trapped in biologically inactive compartments, at least at the time points tested. Furthermore, the observed co-localization with EEA1 (early endosome) and lysosomal associated membrane protein 1 (LAMP1; lysosomal marker) shows very different spatial distribution patterns. hsiRNAs seem to be surrounded within EEA1-enriched endosomes, whereas with LAMP1, we observed partial co-localization where the vesicles enriched with siRNAs and LAMP1 seem to be stacking on each other with partial intersect.

Decreasing the Overall PS Content Decreases Cellular Uptake and Silencing Activity of Hydrophobically Modified siRNAs

Using this panel of hsiRNAs (Figure 1), we next evaluated the impact of oligonucleotide structure and PS content on cellular uptake and silencing activity. The high-PS-content parent configuration showed the highest degree of cellular uptake, consistent with previously reported data (Figure 2).[19] The sense strand alone, which contains both cholesterol and Cy3 label (sense strand only), shows no detectable cellular internalization, confirming our notion that the presence of the guide-strand-derived PS tail is contributing to the efficient uptake. This process relies on both the PS tail and cholesterol, as the oligo lacking cholesterol (no-cholesterol) also shows minimal cellular internalization.

Furthermore, we synthesized three other configurations: 0PS which lacks PS altogether; AS-0PS, which has no PS in the guide strand; and AS-0PS-15-mer, which is a short 15 bp duplex lacking PS in the guide strand. All three configurations, which all lack PS-modified tails, showed limited cellular uptake but at higher levels than the uptake observed for sense strand alone and no-cholesterol-containing configurations. Representative images for the internalization kinetic of the siRNA variants are shown in Figure 2A. To ensure consistency of the observed phenomena, we quantified cellular fluorescence for 75–100 individual images per oligo per time point and repeated these experiments at least twice (see Materials and Methods). The summary of the data is shown in Figure 2B, quantitatively supporting the visual observation that the presence of both the PS tail and cholesterol are required for efficient cellular uptake ($p < 0.0001$, two-way ANOVA for all hsiRNAs compared to the parent).

To evaluate how the observed decrease in uptake impacted silencing efficacy, HeLa cells were treated with hsiRNA variants at seven concentrations to establish half maximal inhibitory concentration (IC_{50}) values for gene silencing. For this experiment, we used a previously validated siRNA sequence targeting the *huntingtin* (*HTT*) gene.[25] Consistent with the observed significant reduction in cellular internalization, *HTT* silencing was substantially reduced with the oligos lacking PS tails, showing close to two orders of magnitude reduction in IC_{50} values (parent = 20 nM, 0PS = 697 nM, AS-0PS = 1,046 nM, AS-0PS-15-mer = 1,378 nM; see Table 1). To verify that the decreased silencing activity was due to reduction in cellular uptake and not efficiency of RISC loading, the same panel was introduced into the cells by lipofection, removing the requirement for self-internalization. All

Figure 1. Structures and Chemical Modification Patterns of Self-Delivering siRNA Compounds Used in the Study
(A) Panel of hsiRNAs used in this study divided into four categories based on phosphorothioate (PS) content and position. Chemical modifications include alternating 2′-O-methyl/2′-fluoro, PS backbone, and cholesterol covalently linked at the 3′ end of the sense strand by a tetraethylene glycol (TEG) linker. (B) Top row: COS-7 cells transiently expressing GFP-EEA1 were treated with 0.25 μM hsiRNA and fixed after 3 h. Middle and bottom rows: HeLa cells treated with 0.25 μM hsiRNA and immunostained with either calnexin (middle row) or LAMP1 (bottom row) and fixed after 24 h. All cells were imaged on a confocal microscope or structured illumination microscope (SIM). Scale bars, 10 μm or 1 μm for insets.

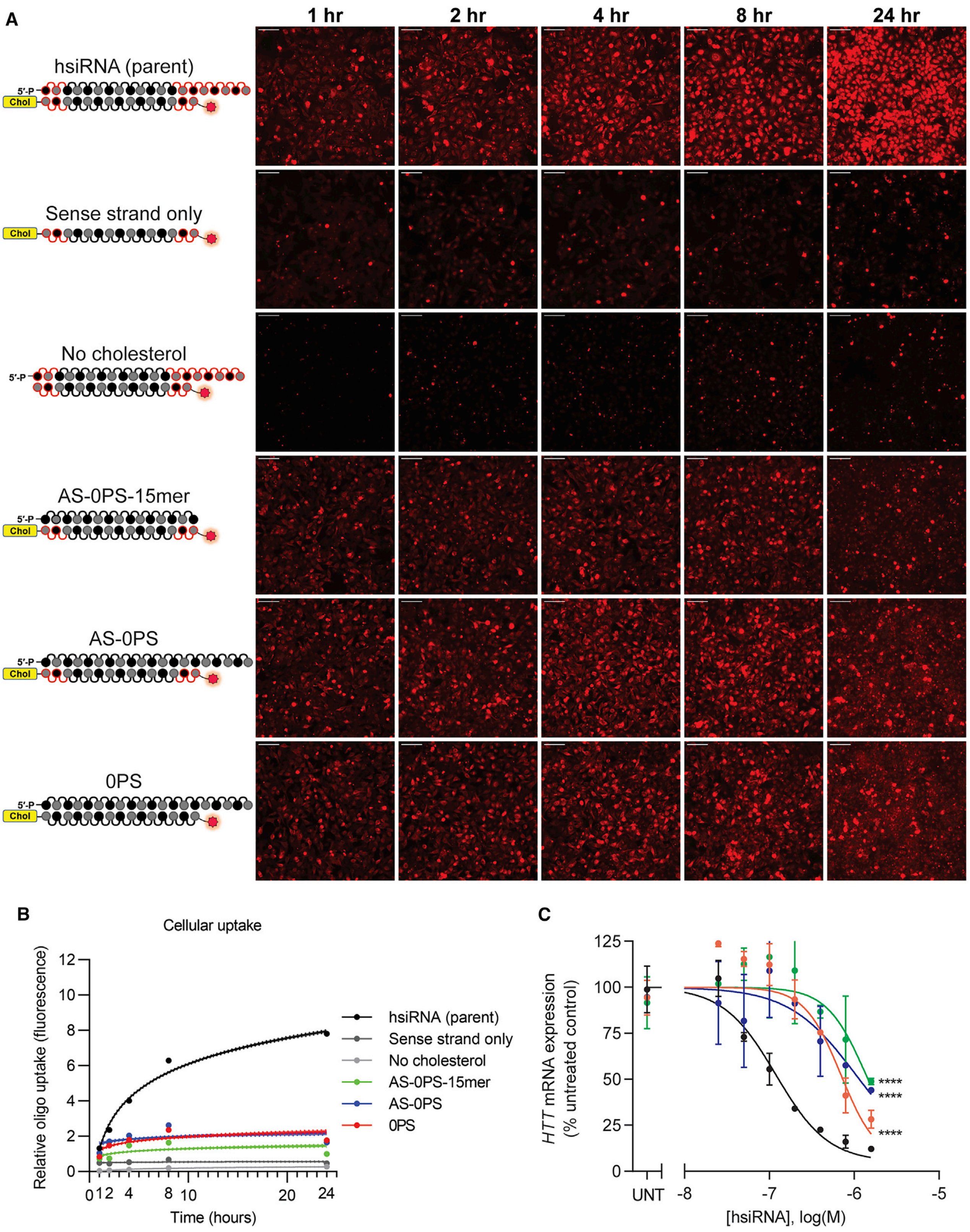

(legend on next page)

Table 1. Summary of hsiRNA Cellular Uptake and Silencing Efficacy

hsiRNA	No. of PS at AS 3′ End	No. of PS on AS 3′ Overhang (PS Tail)	Cellular Uptake (Slope of Semi-log Fit)	No Transfection Reagent (Self-Delivery) IC_{50} (nM)	Lipid-Mediated Transfection IC_{50} (pM)
hsiRNA (parent)	7	5	4.883	20	7.1
0PS	0	0	0.767	697	2.6
AS-0PS	0	0	0.431	1,046	2.1
AS-0PS-15-mer	0	0	0.431	1,378	2,874
Sense strand only	N/A	N/A	0.013	ND	ND
No cholesterol	7	5	0.004	ND	ND
AS-5PS-tail	5	5	3.070	19	2.8
AS-3PS-tail	3	3	2.451	64	3.7
AS-1PS-tail	1	1	1.515	101	1.7
AS-7PS-idT	7 + idT	5	4.050	223	70
AS-1PS-idT	1 + idT	1	0.962	229	53
20-20-mer	7	0	1.626	18	<1
20-18-mer	7	2	2.006	15	<1
sFLT	7	5	7.069	ND	ND
HTT15/sFLT5	7	5	5.480	ND	ND
sFLT15/HTT5	7	5	3.960	ND	ND

Comparison and quantification of all hsiRNAs used in this study. HeLa cells were treated with hsiRNA and mRNA levels were measured 72 h later. N/A, not applicable; ND, not determined.

compounds (except sense strand only and the 15-mer duplex, which are not expected to efficiently enter into RISC as well)[21,26–28] were able to silence mRNA just as well as the parent sequence, indicating that the change in self-delivering silencing efficiency is mainly driven by disruption of self-internalization (Figure S1).

Phosphorothioate-Mediated Antisense Strand Terminal Stabilization, but Not Enhanced Cellular Uptake, Contributes to Productive Silencing Efficacy

We clearly demonstrated that the presence of the PS tail enhances cellular internalization of cholesterol-modified siRNAs and corresponding silencing efficacy. While PS modifications are expected to enhance cellular receptor interaction and thus uptake, they also significantly enhance siRNA stability by providing protection from 3′ to 5′ nucleases.[13] To deconvolute the potential dual roles of PS, we synthesized two other hsiRNA variants where the 3′ ends of the antisense strands were protected from exonuclease degradation by terminal incorporation of inverted dT (idT) in combination with a single terminal PS modification (AS-1PS-idT) (Figure 3A). This configuration is expected to provide significant 3′ to 5′ stabilization to the antisense strand in the absence of extensive PS modifications.[29] As a control, the parent oligo with the terminal idT modification was also synthesized with the number of PSs unchanged (AS-7PS-idT).

AS-7PS-idT showed only slightly decreased internalization efficiency compared to the parent compound, while reduction of PS modifications from seven to one (AS-1PS-idT) resulted in a significant reduction of cellular uptake ($p < 0.0001$, two-way ANOVA for all hsiRNAs compared to the parent), similar to that of the no-PS-containing compound previously tested (Figure 3B). However, despite the difference in PS content, both 3′ idT-modified variants (AS-7PS-idT and AS-1PS-idT) showed identical silencing efficacy that was approximately an order of magnitude less than the parent oligo containing high PS (IC_{50} values: parent = 20 nM, AS-7PS-idT = 223 nM, AS-1PS-idT = 229 nM, see Table 1; Figure 3C). Given that idT-stabilized reduced PS content oligos showed significantly lower uptake, this was highly surprising, indicating that the fraction of internalized compound available for RISC loading was actually higher for the low-PS configuration (AS-1PS-idT).

To evaluate if the observed decrease in activity compared to the parent is due to the negative impact of terminal idT incorporation on RISC assembly, the compounds were introduced into the cells

Figure 2. Decreasing the Overall PS Content Decreases Cellular Uptake and Silencing Activity of Hydrophobically Modified siRNAs

(A) HeLa cells were treated with different hsiRNAs (0.25 μM), fixed at the indicated time points, and imaged on widefield fluorescence microscopy (n = 75–100 images per hsiRNA per time point; see Materials and Methods). Scale bars, 100 μm. (B) Quantification of cellular uptake kinetic from (A) fit to a semi-log line. Shaded area indicates 95% confidence interval. (C) Seven-point dose response performed in HeLa cells. *HTT* mRNA levels were quantified by QuantiGene Assay (Thermo Fisher) after 72 h treatment with hsiRNA. Representative data shown as mean ± SD (n = 3 technical replicates) from at least two independent experiments. UNT, untreated. ****$p < 0.0001$ (two-way ANOVA, compared to hsiRNA [parent]).

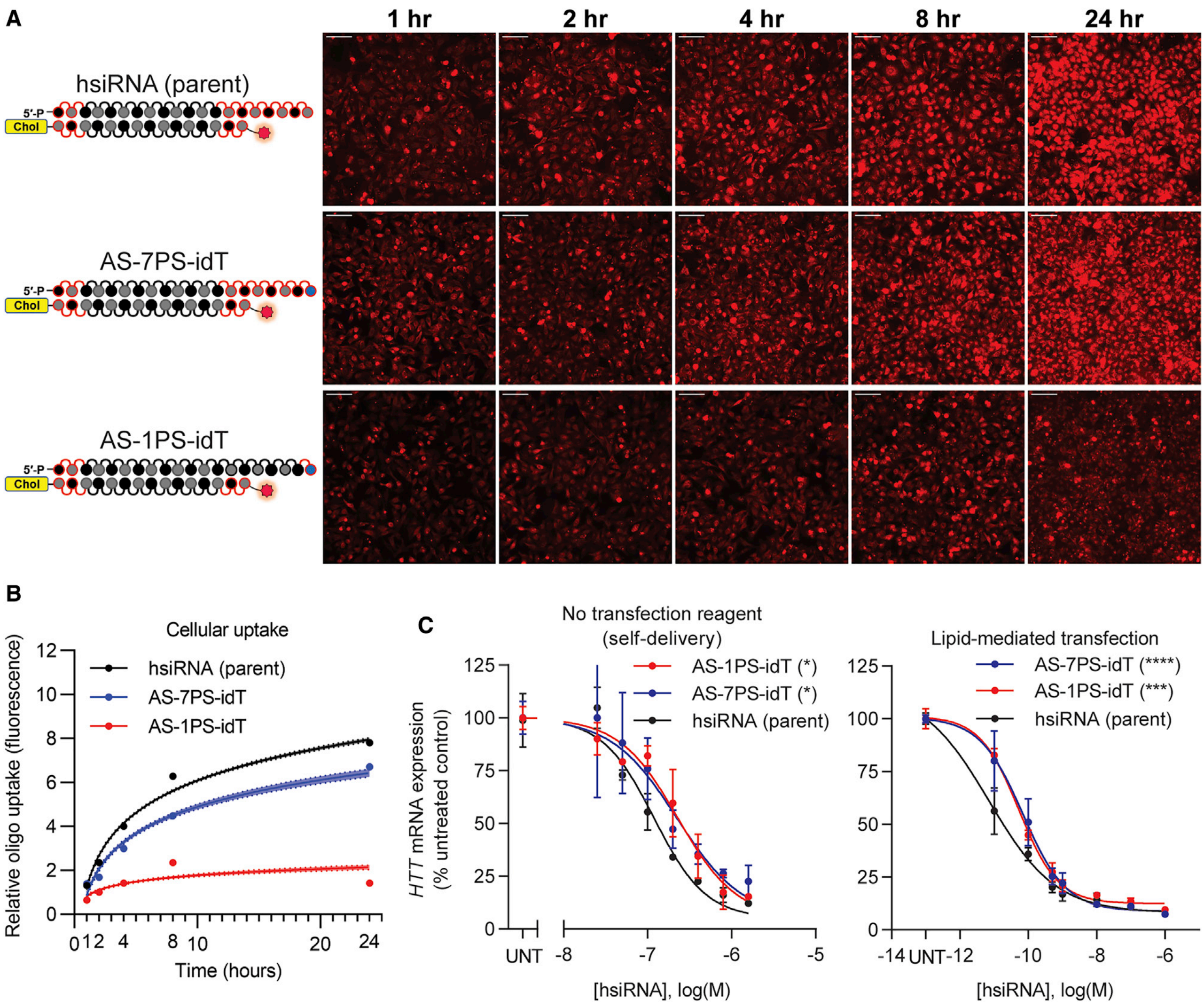

Figure 3. Phosphorothioate-Mediated Antisense Strand Terminal Stabilization, Not Enhanced Cellular Uptake, Contributes to Productive Silencing Efficacy
(A) HeLa cells were treated with different hsiRNAs (0.25 μM), fixed at the indicated time points, and imaged on widefield fluorescence microscopy (n = 75–100 images per hsiRNA per time point; see Materials and Methods). Scale bars, 100 μm. (B) Quantification of cellular uptake kinetic from (A) fit to a semi-log line. Shaded area indicates 95% confidence interval. (C) Seven-point dose response performed in HeLa cells. *HTT* mRNA levels were quantified by QuantiGene Assay (Thermo Fisher) after 72 h treatment with hsiRNA. Representative data shown as mean ± SD (n = 3 technical replicates) from at least two independent experiments. hsiRNA (parent sequence) data are the same data in Figure 2 and are shown again for reference. UNT, untreated. *$p < 0.05$, ***$p < 0.001$, ****$p < 0.0001$ (two-way ANOVA, compared to hsiRNA [parent]).

by lipofection (Figure 3C). Similarly, the idT-modified configurations showed up to 10-fold lower silencing activity compared to the parent compound (IC_{50} values: parent = 7.0 pM, AS-7PS-idT = 70 pM, AS-1PS-idT = 53 pM; see Table 1). Thus, the observed decrease in silencing efficacy is likely due to 3′ idT interference with the PAZ domain of Argonaute 2 (AGO2) interactions, which heavily involve sugar stacking in the correct orientation,[30] one of the major contributing factors for productive RISC assembly.[31–33] Together, these results support the notion that the presence of a single-stranded PS tail enhances gross, but not productive, uptake of siRNAs, and the stabilizing feature of PS modification is a significant contributing factor for efficient mRNA silencing.

Five Terminal Phosphorothioates Are Sufficient to Support Fully Productive Silencing

Given that the PS-mediated antisense 3′ end stabilization is important for activity, we next decided to titrate the number of PS tail modifications sufficient to maintain productive silencing. We synthesized a panel of compounds with different numbers of PS modifications in the tail: seven PSs (parent), five PSs (AS-5PS-tail), three PSs (AS-3PS-tail), and one PS (AS-1PS-tail). As expected, reducing the number of PS modifications had a profound negative impact on cellular internalization (Figure 4A). The compounds containing one or three PS modifications showed background (similar to no PS) levels of cellular uptake. The compound with five PS modifications showed

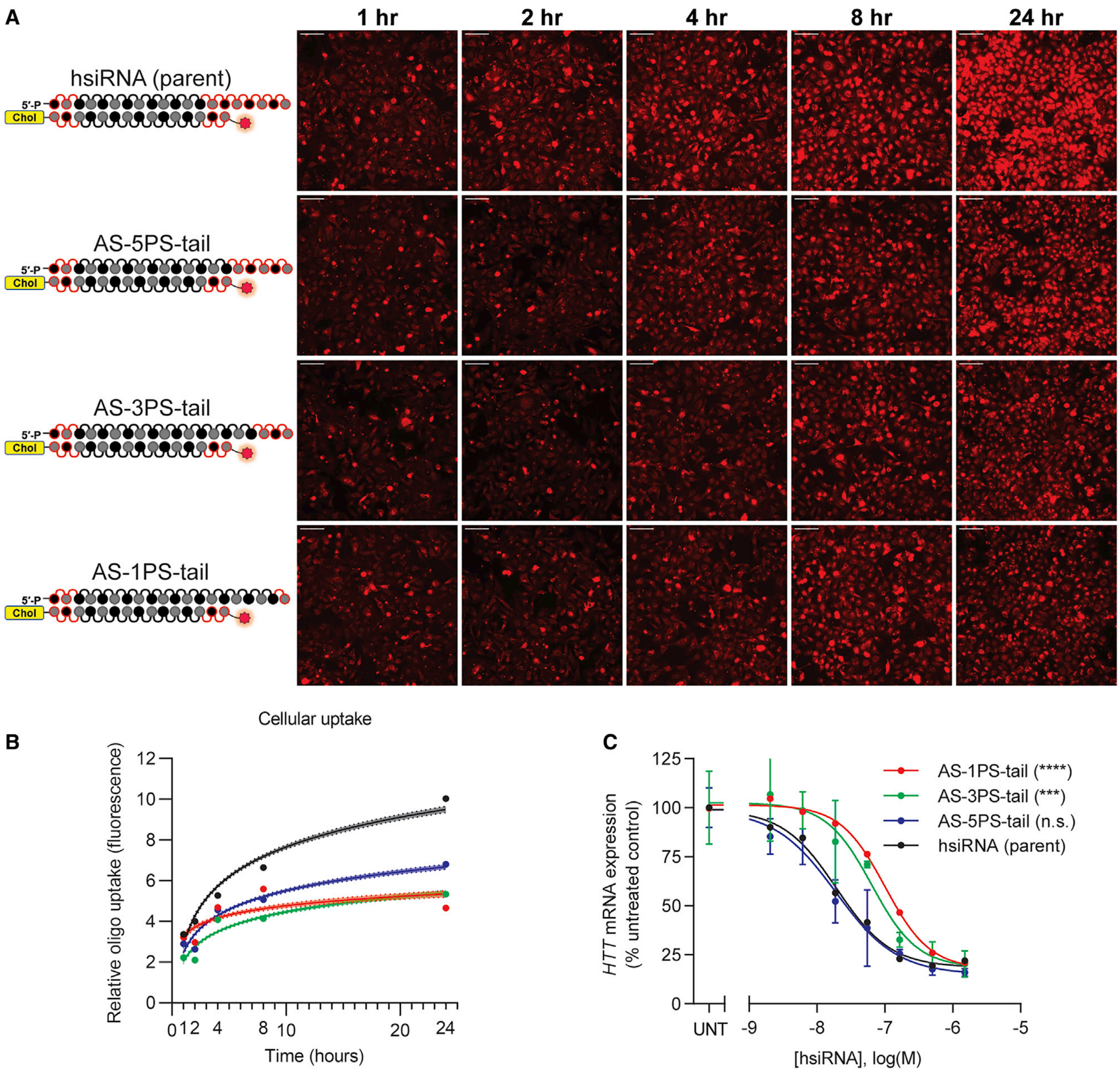

Figure 4. Five Terminal Phosphorothioates Are Sufficient to Support Fully Productive Silencing

(A) HeLa cells were treated with different hsiRNAs (0.25 μM), fixed at the indicated time points, and imaged on widefield fluorescence microscopy (n = 75–100 images per hsiRNA per time point; see Materials and Methods). Scale bars, 100 μm. (B) Quantification of cellular uptake kinetic from (A) fit to a semi-log line. Shaded area indicates 95% confidence interval. (C) Seven-point dose response performed in HeLa cells. *HTT* mRNA levels were quantified by QuantiGene Assay (Thermo Fisher) after 72 h treatment with hsiRNA. Representative data shown as mean ± SD (n = 3 technical replicates) from at least two independent experiments. hsiRNA (parent sequence) data are the same data in Figure 2 and are shown again for reference. UNT, untreated, n.s., not significant. ***p < 0.001, ****p < 0.0001 (two-way ANOVA, compared to hsiRNA [parent]).

cellular internalization in between the parent and background levels (Figure 4B, $p < 0.0001$, two-way ANOVA for all hsiRNAs compared to the parent). These results indicate that at least five PS backbones are necessary to promote efficient cellular internalization, consistent with data reported for ASOs, where relatively long stretches of PS modifications were shown to be necessary to impact tissue distribution and efficacy.[34]

When we measured silencing activity, the five-PS-containing compound was similar to the parent (IC_{50} values: parent = 20 nM, AS-5PS-tail = 19 nM; see Table 1), while AS-3PS-tail and AS-1PS-tail siRNAs demonstrated an ~5-fold reduction in efficacy (IC_{50} values: 64 nM, 101 nM, respectively; see Table 1). Again, when these compounds were transfected by lipofection, they all demonstrated very similar IC_{50} values all in the single-digit picomolar range, indicating

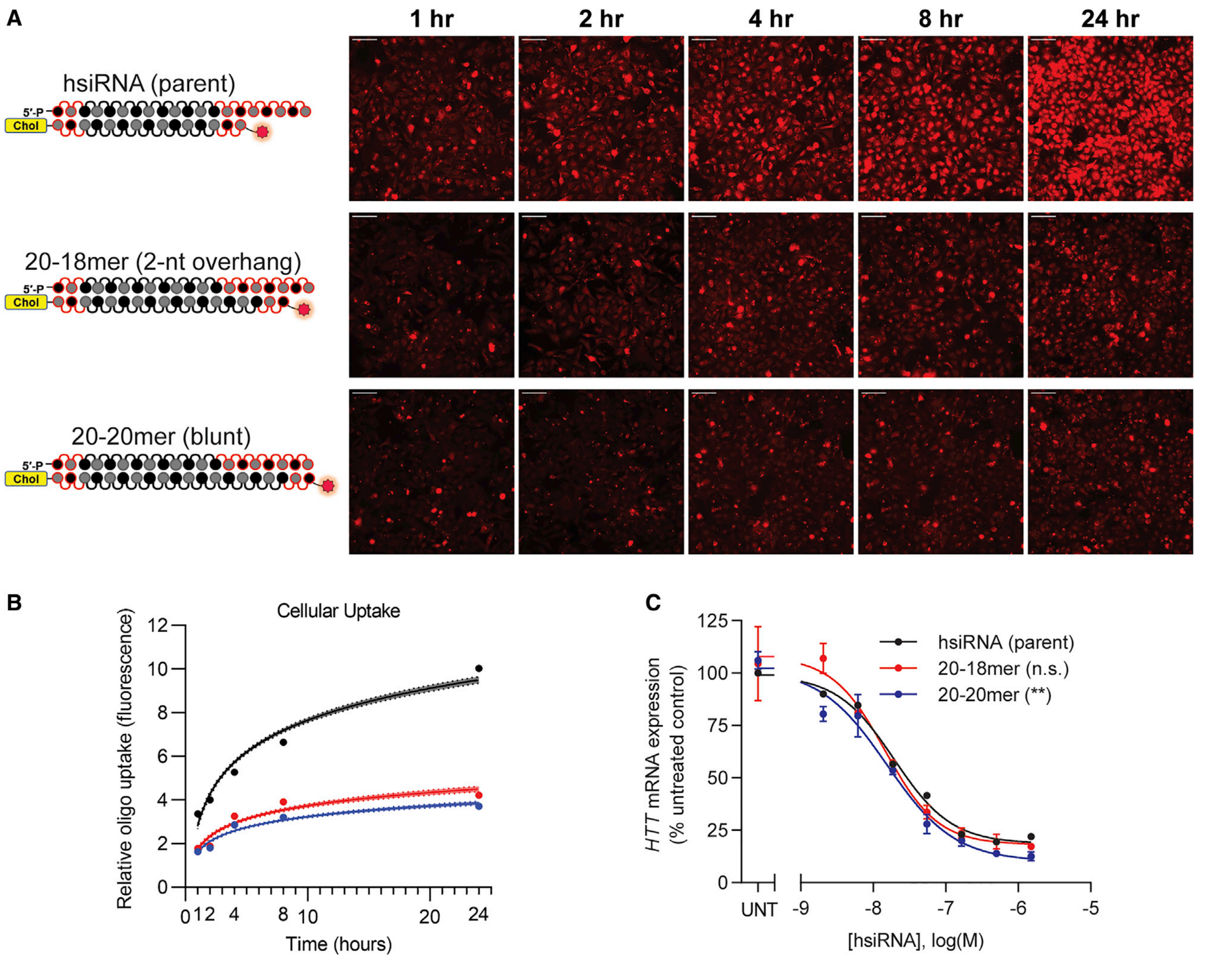

Figure 5. Phosphorothioate-Mediated Enhancement of Cellular Uptake Is Only Observed in a Single-Stranded, but Not Double-Stranded, Context

(A) HeLa cells were treated with different hsiRNAs (0.25 μM), fixed at the indicated time points, and imaged on widefield fluorescence microscopy (n = 75–100 images per hsiRNA per time point; see Materials and Methods). Scale bars, 100 μm. (B) Quantification of cellular uptake kinetic from (A) fit to a semi-log line. Shaded area indicates 95% confidence interval. (C) Seven-point dose response performed in HeLa cells. *HTT* mRNA levels were quantified by QuantiGene Assay (Thermo Fisher) after 72 h treatment with hsiRNA. Representative data shown as mean ± SD (n = 3 technical replicates) from at least two independent experiments. hsiRNA (parent sequence) data are the same data in Figure 2 and are shown again for reference. UNT, untreated; n.s., not significant. **$p < 0.01$ (two-way ANOVA, compared to hsiRNA [parent]).

that the observed differences are not due to RISC loading but instead are in their cellular uptake efficiency (Figure S2). Taken together, these data indicate that the presence of five, but not three or fewer, PS modifications was sufficient to provide the required stabilization for efficient self-delivering siRNA silencing activity.

Phosphorothioate-Mediated Enhancement of Cellular Uptake Is Only Observed in a Single-Stranded, but Not Double-Stranded, Context

The geometry of a single-stranded and double-stranded backbone presentation to the biological environment is significantly different.[35] Because the presence of the single-stranded PS-modified tail significantly enhances cellular internalization, we decided to evaluate the relative impact of different hsiRNA structural configurations on PS-mediated uptake (Figure 5A). We synthesized a panel of siRNAs that share the same antisense strand containing seven PS terminal modifications. The identical antisense strand was annealed to sense strands of varying lengths (15-, 18-, and 20-nt long), generating compounds with identical PS compositions in different structural contexts (single-stranded regions of 5-nt, 2-nt, and 0-nt [blunt]).

Enhanced cellular uptake was only observed in the context of an asymmetric compound, while the blunt (20-20-mer) and 2-nt tail (20-18-mer) variants showed low levels of internalization similar to the no-PS compounds ($p < 0.0001$, two-way ANOVA for all hsiRNAs compared to the parent). These data indicate that only PS modification

in the single-stranded but not double-stranded context contributes to enhanced cellular internalization. Despite the fact that the cellular uptake of the non-asymmetric compounds was reduced (Figure 5B), the silencing activity was again comparable to the parent sequence at the time point tested (Figure 5C), consistent with the notion that PS-mediated enhancement of cellular internalization is not contributing to productive uptake, at least short term. As a control, these compounds were also transfected via lipofection and showed very similar IC_{50} values in the single-digit picomolar range (Figure S3).

The Sequence of the Single-Stranded Phosphorothioate Tail Affects Cellular Uptake

We have clearly demonstrated that both the structural and chemical configuration of the siRNA may have a profound impact on cellular internalization. All previous experiments were done using an siRNA sequence targeting *HTT*.[25] In the context of ASOs, the PS-mediated cellular uptake and tissue distribution may be heavily affected by the sequence, as PS-mediated interactions with cell surface and intracellular proteins are sequence-context dependent.[36]

As the enhanced cellular uptake of hydrophobic siRNA is dependent on the presence of a single-stranded PS tail similar to a PS-containing ASO, we decided to evaluate if the sequence context of the tail may also have an impact on efficiency of cellular internalization. When two different siRNAs, one targeting *sFLT* ($siRNA^{sFLT}$) and another targeting *HTT* ($siRNA^{HTT}$)[37] were compared, we found that $siRNA^{sFLT}$ consistently showed higher cellular internalization (Figure 6A, $p < 0.0001$, two-way ANOVA). To ensure that this effect was not due to a batch effect of oligonucleotide synthesis, we re-synthesized both siRNAs in parallel using the same reagents and observed the same results (data not shown).

To test if the observed differences in efficiency of cellular uptake was due to the sequence of the single-stranded phosphorothioated 3′ tail, we synthesized a panel of chimeric sequences where the 5-nt tail was swapped between $siRNA^{HTT}$ (AUAUA) and $siRNA^{sFLT}$ (GAGAG) to make $siRNA^{HTT15/sFLT5}$ and $siRNA^{sFLT15/HTT5}$ (Figure 6A). As expected, incorporation of the "high activity" sFLT tail in the context of the HTT sequence ($siRNA^{HTT15/sFLT5}$) compound significantly enhances its uptake. Likewise, changing the sFLT1 tail to the "low activity" HTT tail resulted in a significant decrease in cellular internalization ($p < 0.0001$, two-way ANOVA for all hsiRNAs compared to the parent). Thus, sequence of the single-stranded PS tail may contribute to the efficiency of hydrophobic siRNA cellular internalization.

DISCUSSION

Conjugate-mediated siRNA delivery is being realized as the clinically dominant technology, replacing lipid nanoparticle (LNP) encapsulation, which was explored initially.[2,38] Although the mechanism behind LNP-mediated uptake is relatively well characterized,[16,39–42] there is little known about the mechanism behind conjugate-mediated internalization. A range of different conjugates have been explored *in vivo*: GalNAc delivers specifically to hepatocytes, while hydrophobic modifications enable delivery to a wide range of tissues beyond the liver.[5,43,44]

We have previously demonstrated that the presence of PS modifications enhances siRNA *in vivo* accumulation with both hydrophobic and GalNAc conjugates.[20] In both research[13] and clinical settings,[1] phosphorothioates are one of the most commonly used modifications. While it is clear that PS chemistry is fundamental for ASO *in vivo* activity and cellular distribution, with significant research dedicated toward detailed understanding of this process,[36] the functional contribution of PS on siRNA delivery has never been systematically characterized.

In this study, we used a fully chemically stabilized, asymmetric cholesterol-conjugated siRNA configuration as a model. A combination of a longer guide strand (20-nt) and shorter sense strand (15-nt) generates a compound with a fully phosphorothioated single-stranded tail, which impacts the efficiency of cellular uptake in cultured cells and *in vivo*.[45] We systematically evaluated the impact of structure, number, and sequence context of the PS tail on cellular internalization and efficacy. We demonstrate that both structural context (single-strand versus double-stranded), number of PS backbones, and sequence have an impact on the efficiency of cellular uptake. Surprisingly, we found a lack of correlation between the observed enhancement of cellular internalization and productive silencing efficacy, likely indicating that PS-mediated uptake mostly drives compounds into non-productive "sinks," a phenomenon that has previously been described both for ASOs[46,47] and siRNAs.[24,48] We also demonstrate that complete dependence of productive silencing in the presence of reduced numbers of PS modifications is likely due to these modifications contributing to oligonucleotide stability and not internalization.

It has long been believed that the majority of internalized oligonucleotides are trapped non-productively in endosomes and lysosomes, with only a small fraction of compounds becoming biologically active.[24,40,49] Here we show the significant decrease in the amount of internalized low-PS-content siRNA compared to high-PS-content compounds. Surprisingly, at least at short time points in cultured cells (72 h), there is no difference in silencing IC_{50} values, indicating that a relatively higher fraction of low-PS-content siRNAs is immediately functionally available. While PS modifications have a minimal impact on the overall shape of the oligonucleotide, they have a profound impact on the backbone's chemical and functional properties, resulting in increased affinity to both gross and selective protein interactions.[13] These additional contacts are likely to contribute to the observed enhancement in cellular uptake. It is also possible that PS-driven diversification of intracellular interactions might compete with siRNA availability for RISC assembly, resulting in a smaller fraction of the intracellular oligonucleotides becoming functionally active. These interactions might be related to the efficiency of endosomal escape or other mechanisms. This paper focuses on characterization in cultured cells and shows that PS-derived enhancement in productive uptake is not biologically significant. More studies are necessary to understand the functional impact of PS-mediated

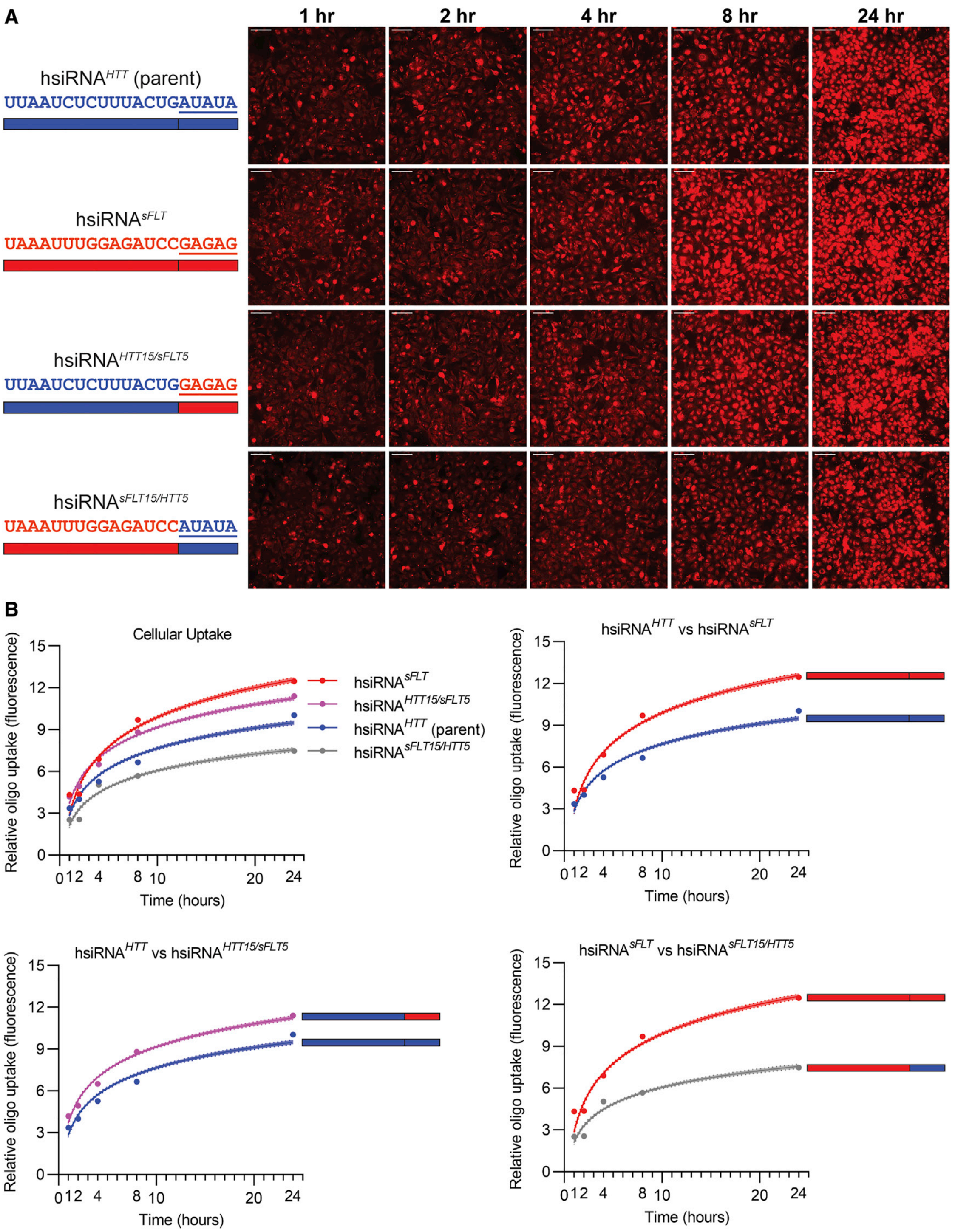

Figure 6. The Sequence of the Single-Stranded Phosphorothioate Tail Affects Cellular Uptake

(A) HeLa cells were treated with different hsiRNAs (0.25 μM), fixed at the indicated time points, and imaged on widefield fluorescence microscopy (n = 75–100 images per hsiRNA per time point; see Materials and Methods). hsiRNA (parent sequence) data are the same data in Figure 2 and are shown again for reference. Scale bars, 100 μm. (B) Quantification of cellular uptake kinetic from (A) fit to a semi-log line. Shaded area indicates 95% confidence interval. Top left panel shows hsiRNA^{HTT} and hsiRNA^{sFLT} and their chimeric tail-swapped sequences, hsiRNA$^{HTT15/sFLT5}$ and hsiRNA$^{sFLT15/HTT5}$. Other panels compare only two hsiRNAs compared at a time for clarity, showing that hsiRNA^{sFLT} tail sequence enhances cellular uptake compared to the hsiRNA^{HTT} tail sequence.

enhancement of cellular accumulation on long-term silencing (months), which will need to be evaluated separately in the context of each conjugate and tissue.

Cholesterol-conjugated siRNAs without any PS modifications fully lose silencing efficacy. We show that introduction of a 3′ idT at the 3′ end of the antisense strand in the context of a single PS modification allows the compound to regain efficacy close to the idT-modified, high-PS-content control (seven PS), indicating that the primary contributing factor of the terminal PS modification is enhancement of stability. With that said, introduction of the idT results in decreased silencing efficacy by more than an order of magnitude relative to the parent compound, both in self-delivering and lipid-mediated uptake. While idT is one of the modifications previously used for 3′ guide strand stabilization,[50] it is clearly not optimal. The negative impact on efficacy is likely due to disruption of proper PAZ domain interactions with the antisense strand's terminal bases that normally contribute to efficient RISC assembly. Evaluation of the published PAZ domain crystal structure[51,52] in complex with an antisense strand shows multiple interactions with the two terminal nucleotides, which will all be naturally distorted in the context of an idT. Conversely, several terminal PS modifications resulted in similar stabilization but did not negatively affect RISC assembly.

In the context of ASOs, PS modifications are one of the primary chemical modifications driving both distribution as well as toxicity through a variety of possible mechanisms.[15,16] The different types of toxicities include enhanced alanine transaminase (ALT), thrombocytopenia, inflammation, hepatotoxicity, and nephrotoxicity.[53,54] Recently, compounds with mixed backbones (phosphorothioate and phosphodiester) are gaining traction by demonstrating a better ratio between efficacy and toxicity. For example, in CNS, clinically advanced ASO utilizes a mixed backbone configuration.[55] Naturally, minimization of PS content might be considered to enhance safety of therapeutic siRNAs. In cultured cells, five terminal PS backbones were sufficient to support full silencing capacity. *In vivo*, the exact number and location of PS backbones will need to be optimized for each conjugate modality individually because of the complexity of competing biological interactions oligonucleotides that are involved; the compounds need to be stable, bind to serum proteins, associate with cellular surfaces, escape endosomes, etc. Thus, the optimal configuration in cultured cells might not be translatable for *in vivo* use.

With ASOs, sequence is one of the critical factors affecting a compound's efficacy and distribution, even in a context of identical chemical modification patterns.[1,15] This is driven by a combination of aptameric effects as well as sequence and chemical modifications all contributing to PS-driven protein interactions. However, the relative contribution of sequence is less in the context of siRNAs due to the rigidity of its double-stranded helix structure. With that said, in the context of asymmetric siRNAs that contain a small single-stranded PS-modified region, this tail might be contributing to the efficiency of cellular internalization. While the sequence impact is less profound compared to the number and location of PS modifications, it is still measurable and reproducible. The first possible explanation for the observed difference in sequences is change in stability. We tested this hypothesis but did not detect any measurable changes (data not shown). It is more likely that the sequence context of the PS tail may be affecting efficiency of cellular interactions similar in mechanism to those reported for ASOs.[15] There are multiple articles describing functional pathways involved in productive internalization of ASOs inside the cells; while many protein co-factors are described (for example, lysobisphosphatidic acid,[56] Sec31A,[57] and Rab5[58]), it is clear that productive interactions are redundant and knockout of any of the aforementioned pathways individually does not fully block productive silencing. Thus, complete functional characterization of molecular mechanisms behind PS-enhanced uptake is technically complex and will require further investigation.

In conclusion, here we present a systematic analysis of the relative PS-modified backbone contribution to conjugate-mediated uptake of siRNAs and show that PS enhancement of cellular internalization might not functionally contribute to RNAi-mediated silencing, at least in cultured cells.

MATERIALS AND METHODS

Oligonucleotide Synthesis, Deprotection, and Purification

All oligonucleotide sequences are listed in Table S1. Oligonucleotides were synthesized using modified (2′-F, 2′-OMe, 3′ idT) phosphoramidites with standard protecting groups. Solid-phase synthesis conditions using a MerMade 12 (BioAutomation) or Dr Oligo 48 (Biolytic) were performed using modified protocols. Unconjugated oligonucleotides were grown on controlled pore glass functionalized with a long-chain alkyl amine and unylinker terminus (Chemgenes). Cholesterol-conjugated oligonucleotides were synthesized on controlled pore glass functionalized with succinyl tetraethylene glycol (TEG) linker and cholesterol terminus (Chemgenes). Cy3 dye (Quasar 570 CE) phosphoramidite was purchased from (GenePharma). Phosphoramidites were prepared at 0.1 M in anhydrous acetonitrile (ACN), with added dry 15% dimethylformamide (DMF) in the 2′-OMe U amidite. 5-(Benzylthio)-1H-tetrazole (BTT) was used as the activator at 0.25 M. Detritylations were performed using 3% trichloroacetic acid in dichloromethane. Capping was done with non-tetrahydrofuran-containing reagents CAP A, 20% *n*-methylimidazole in ACN and CAP B, 20% acetic anhydride (Ac_2O), 30% 2,6-lutidine in ACN (Synthesis reagents were purchased at AIC). Sulfurization was performed with 0.1 M solution of 3-[(dimethylaminomethylene)amino]-3H-1,2,4-dithiazole-5-thione (DDTT) in pyridine (ChemGenes) for 3 min. Phosphoramidite coupling times were 3 min for all amidites used.

Deprotection and Purification of Oligonucleotides

Both sense and antisense strands were cleaved and deprotected using 40% aqueous methylamine at 45°C for 60 min. The oligonucleotide solutions were then cooled in a freezer for a few minutes and filtered to remove the control pore glass from the cleaved oligo. The filtrate was cooled with dry ice and then dried under vacuum in a Speedvac.

The resulting pellets were re-suspended in 5% ACN in water. The purification of antisense strands was performed on an Agilent 1100 series system, equipped with an Agilent PL-SAX, polymer ion exchange column (10 × 100 mm), or Source Q anion exchange resin (GE Healthcare) (10 × 100 mm custom-packed column) using the following conditions: eluent A, 20% ACN, 20 mM NaAc (pH 5); eluent B, 1 M sodium perchlorate in 20% ACN; gradient, 0% B 2 min, 35% B 12 min, clean and re-equilibration to initial conditions 6 min. Purification of sense strands was performed on the same equipment but equipped with a PRP-C18, a polymer reverse phase column (10 × 100 mm), using the following conditions: eluent A, 50 mM sodium acetate in 5% ACN; eluent B, ACN; gradient, 0% B 2 min, 0%–40% B 1 min, 40%–70% B 9 min, clean and re-equilibration 6 min. Temperature, 70 °C, and flow rate, 10 mL min^{-1}, were the same in both cases. Peaks were monitored at 260 nm, and 550 nm for labeled oligonucleotides. The pure oligonucleotide fractions were collected, individually characterized by liquid chromatography-mass spectrometry (LC-MS), combined, frozen, and dried in a Speed-vac overnight. Oligonucleotides were re-suspended in 5% ACN, desalted through fine Sephadex G-25 (GE Healthcare) (15 × 200 mm custom-packed column), and lyophilized. All reagents mentioned above were purchased from Sigma Aldrich and used as per manufacturer's instructions, unless otherwise stated.

LC-MS Analysis of Oligonucleotides

The identity of oligonucleotides was established by LC-MS analysis on an Agilent 6530 accurate mass Q-TOF LC-MS machine using the following conditions: buffer A: 100 mM hexafluoroisopropanol/9 mM triethylamine in LC-MS grade water; buffer B: 100 mM hexafluoroisopropanol/9 mM triethylamine in LC-MS grade methanol; column, Agilent AdvanceBio oligonucleotides C18; gradient antisense, 0% B 1 min, 0%–30% B 8 min, clean and re-equilibration 4 min; gradient sense, 0% B 1 min, 0%–50% B 0.5 min, 50%–100% B 8 min, clean and re-equilibration 4 min; temperature, 45°C; flow rate, 0.5 mL min^{-1}, UV (260 nm) and (550 nm for labeled oligonucleotides). MS parameters: source, electrospray ionization; ion polarity, negative mode; range, 100–3,200 *m/z*; scan rate, 2 spectra s^{-1}; capillary voltage, 4,000; fragmentor, 180 V. All reagents mentioned above were purchased form Sigma Aldrich and used as per manufacturer's instructions, unless otherwise stated.

Cell Culture and Transfection

Both HeLa and COS-7 cells were maintained in DMEM (Invitrogen) supplemented with 100 U/mL penicillin streptomycin (Invitrogen), and 10% fetal bovine serum (FBS) (Atlanta Biologicals) at 37°C and 5% CO_2. For GFP-EEA1 transfections, calcium phosphate was used to transfect 1 μg DNA.[11] The cells were then washed with fresh media after 16 h and allowed to grow for an additional 24 h.

Cellular Uptake

HeLa cells were seeded at a density of 200,000 cells/well in 12-well glass-bottom plates (MatTek P12G-1.5-10-F) in DMEM and 3% FBS and grown overnight. The next day, the cells were treated with 0.25 μM siRNA in fresh media. At different time points (1, 2, 4, 8, or 24 h), the cells were washed twice with ice-cold PBS and then fixed in 10% neutral-buffered formalin for 10 min at room temperature (RT) (Sigma HT501128-4L). The cells were then washed three times with PBS for 5 min each and then stained with 4′,6-diamidino-2-phenylindole (DAPI) (Thermo Fisher D1306) or Hoechst 33342 (Thermo Fisher H3570). Fluorescence microscopy images were acquired with a Leica DMi8 widefield fluorescence microscope using the Navigator module to take 75–100 images per well. After image acquisition, images were exported in 16-bit tagged image file format (TIFF) format from the Leica LAS X software and processed in Fiji v1.52p to quantify the kinetics of siRNA cellular uptake by measuring the mean intensity for the Cy3 channel and normalizing to the number of cells per image.[59–61] The kinetic was plotted in GraphPad Prism 8 software and fit to a semi-log line.

Immunofluorescence

HeLa cells were seeded at a density of 2 × 10^5 cells in 35 mm dishes (MatTek P35G-0.170-14-C) in DMEM + 3% FBS and grown overnight. The next day, the cells were treated with 0.25 μM siRNA. At the indicated time points, the cells were fixed as described above, permeabilized for 15 min at RT (0.1% Triton X-100, 0.1% Tween-20 in PBS), washed three times with PBST (0.1% Tween-20 in PBS) for 5 min each, blocked for 1 h at RT (1% wt/vol BSA, 5% goat serum, 0.1% Triton X-100 in PBS), and then washed three times with PBST for 5 min each. The cells were incubated with primary antibody overnight at 4°C, washed three times with PBST for 5 min each, and then incubated with secondary antibody for 1 h at RT and washed three times with PBS for 5 min each. All antibodies were diluted in 1% wt/vol BSA, 0.1% Triton X-100 in PBS. The cells were then stained with DAPI (1 μg/ml) as described above and mounted with VectaShield H-1000 mounting medium. Primary antibodies used were LAMP1 (Cell Signaling 9091S, 1:200) and calnexin (Abcam ab31290, 1:1000). Secondary antibody used was anti-rabbit Alexa-Fluor-488 (Invitrogen A-11008, 1:500).

Super-resolution three-dimensional structured illumination microscopy (3D-SIM) images were acquired on a DeltaVision OMX V4 (GE Healthcare) equipped with a 60×/1.42 NA PlanApo oil immersion lens (Olympus); 405, 488, 568, and 642 nm solid-state lasers; and sCMOS cameras (pco.edge). Image stacks of 10 μm with 0.125 μm thick z sections and 15 images per optical slice (3 angles and 5 phases) were acquired using immersion oil with a refractive index of 1.514. Images were reconstructed using Wiener filter settings of 0.008 for the 405 channel, 0.006 for the 528 channel, and 0.003 for the 508 and 683 channels. Optical transfer functions (OTFs) were measured specifically for each channel with SoftWoRx 6.1.3 (GE Healthcare) to obtain super-resolution images with a 2-fold increase in resolution both axially and laterally. Images from different color channels were registered using parameters generated from a gold grid registration slide (GE Healthcare) and SoftWoRx 6.5.2 (GE Healthcare).

Gene Expression Analysis

To measure silencing activity, we performed a 7-point dose response, with concentrations ranging from 0 μM to 1.5 μM, after 72 h. All hsiRNAs use a targeting sequence for huntingtin (*HTT*) mRNA, which has previously been validated.[25] Briefly, HeLa cells, resuspended in DMEM with 6% FBS and without antibiotics, were added into each well of a 96-well plate with a density of 5,000 cells/50 μL per well. hsiRNA was diluted to two times final concentration in serum-free OptiMEM, and 50 μL of compound was added to each well for a final volume of 100 μL/well and a final concentration of 3% FBS. The mixture was incubated under standard conditions for 72 h and then cells were lysed and processed according to the manufacturer's recommended protocol using the Quantigene bDNA Assay 2.0 (Thermo Fisher QS0011) and were normalized to the housekeeping gene *HPRT*. Briefly, cells were lysed in 250 μL diluted lysis mixture (1:2 lysis mixture:water) with 0.167 ug/μL proteinase K (Thermo Fisher QS0103) for 30 min at 55°C. Cell lysates were mixed thoroughly, and 20–80 μL of lysate were added to a bDNA capture plate along with 0–60 additional diluted lysis mixture without proteinase K to fill up to 80 μL total volume. Probe sets were diluted as specified in Thermo Fisher protocol, and 20 μL of either human *HTT* (Thermo Fisher SA-50339) or *HPRT* (Thermo Fisher SA-10030) were added to each well of capture plate to a final volume of 100 μL. Luminescence was detected on a Tecan M 1000.

Statistical Analysis

Data were analyzed using GraphPad Prism 8 software (https://www.graphpad.com/scientific-software/prism/). IC_{50} curves were fitted using log(inhibitor) versus response—variable slope (four parameters). To compare dose-response curves, two-way ANOVA Tukey's test for multiple comparisons compared to the hsiRNA (parent) was used. $p < 0.05$ was considered significant.

SUPPLEMENTAL INFORMATION

Supplemental Information can be found online at https://doi.org/10.1016/j.omtn.2020.07.029.

AUTHOR CONTRIBUTIONS

S.L. and A.K. conceived the experiments and analyzed the data. D.E. and J.S. synthesized the oligonucleotides used. S.L. performed the experiments. S.L. and A.K. prepared the figures and wrote the manuscript.

CONFLICT OF INTEREST

The authors declare no competing interests.

ACKNOWLEDGMENTS

We thank all the members of the Khvorova laboratory for helpful discussions, the Mello laboratory for excellent technical assistance and guidance on confocal microscopy, and the SCOPE core at UMass Medical School for assistance with SIM microscopy. This work was supported by National Institutes of Health grants S10 OD020012 to A.K. and MIRA GM 131839-01 to A.K.

REFERENCES

1. Khvorova, A., and Watts, J.K. (2017). The chemical evolution of oligonucleotide therapies of clinical utility. Nat. Biotechnol. *35*, 238–248.
2. Setten, R.L., Rossi, J.J., and Han, S.P. (2019). The current state and future directions of RNAi-based therapeutics. Nat. Rev. Drug Discov. *18*, 421–446.
3. Adams, D., Gonzalez-Duarte, A., O'Riordan, W.D., Yang, C.C., Ueda, M., Kristen, A.V., Tournev, I., Schmidt, H.H., Coelho, T., Berk, J.L., et al. (2018). Patisiran, an RNAi therapeutic, for hereditary transthyretin amyloidosis. N. Engl. J. Med. *379*, 11–21.
4. Foster, D.J., Brown, C.R., Shaikh, S., Trapp, C., Schlegel, M.K., Qian, K., Sehgal, A., Rajeev, K.G., Jadhav, V., Manoharan, M., et al. (2018). Advanced siRNA Designs Further Improve In Vivo Performance of GalNAc-siRNA Conjugates. Mol. Ther. *26*, 708–717.
5. Alterman, J.F., Godinho, B.M.D.C., Hassler, M.R., Ferguson, C.M., Echeverria, D., Sapp, E., Haraszti, R.A., Coles, A.H., Conroy, F., Miller, R., et al. (2019). A divalent siRNA chemical scaffold for potent and sustained modulation of gene expression throughout the central nervous system. Nat. Biotechnol. *37*, 884–894.
6. Matsuda, S., Keiser, K., Nair, J.K., Charisse, K., Manoharan, R.M., Kretschmer, P., Peng, C.G.V., V Kel'in, A., Kandasamy, P., Willoughby, J.L.S., et al. (2015). siRNA conjugates carrying sequentially assembled trivalent N-acetylgalactosamine linked through nucleosides elicit robust gene silencing in vivo in hepatocytes. ACS Chem. Biol. *10*, 1181–1187.
7. Soutschek, J., Akinc, A., Bramlage, B., Charisse, K., Constien, R., Donoghue, M., Elbashir, S., Geick, A., Hadwiger, P., Harborth, J., et al. (2004). Therapeutic silencing of an endogenous gene by systemic administration of modified siRNAs. Nature *432*, 173–178.
8. Wolfrum, C., Shi, S., Jayaprakash, K.N., Jayaraman, M., Wang, G., Pandey, R.K., Rajeev, K.G., Nakayama, T., Charrise, K., Ndungo, E.M., et al. (2007). Mechanisms and optimization of in vivo delivery of lipophilic siRNAs. Nat. Biotechnol. *25*, 1149–1157.
9. Nikan, M., Osborn, M.F., Coles, A.H., Godinho, B.M., Hall, L.M., Haraszti, R.A., Hassler, M.R., Echeverria, D., Aronin, N., and Khvorova, A. (2016). Docosahexaenoic acid conjugation enhances distribution and safety of siRNA upon local administration in mouse brain. Mol. Ther. Nucleic Acids *5*, e344.
10. Rajeev, K.G., Nair, J.K., Jayaraman, M., Charisse, K., Taneja, N., O'Shea, J., Willoughby, J.L.S., Yucius, K., Nguyen, T., Shulga-Morskaya, S., et al. (2015). Hepatocyte-specific delivery of siRNAs conjugated to novel non-nucleosidic trivalent N-acetylgalactosamine elicits robust gene silencing in vivo. ChemBioChem *16*, 903–908.
11. Ly, S., Navaroli, D.M., Didiot, M.-C., Cardia, J., Pandarinathan, L., Alterman, J.F., Fogarty, K., Standley, C., Lifshitz, L.M., Bellve, K.D., et al. (2017). Visualization of self-delivering hydrophobically modified siRNA cellular internalization. Nucleic Acids Res. *45*, 15–25.
12. Huang, Y. (2017). Preclinical and Clinical Advances of GalNAc-Decorated Nucleic Acid Therapeutics. Mol. Ther. Nucleic Acids *6*, 116–132.
13. Eckstein, F. (2014). Phosphorothioates, essential components of therapeutic oligonucleotides. Nucleic Acid Ther. *24*, 374–387.
14. Bailey, J.K., Shen, W., Liang, X.H., and Crooke, S.T. (2017). Nucleic acid binding proteins affect the subcellular distribution of phosphorothioate antisense oligonucleotides. Nucleic Acids Res. *45*, 10649–10671.
15. Shen, W., De Hoyos, C.L., Migawa, M.T., Vickers, T.A., Sun, H., Low, A., Bell, T.A., 3rd, Rahdar, M., Mukhopadhyay, S., Hart, C.E., et al. (2019). Chemical modification of PS-ASO therapeutics reduces cellular protein-binding and improves the therapeutic index. Nat. Biotechnol. *37*, 640–650.
16. Vickers, T.A., Rahdar, M., Prakash, T.P., and Crooke, S.T. (2019). Kinetic and subcellular analysis of PS-ASO/protein interactions with P54nrb and RNase H1. Nucleic Acids Res. *47*, 10865–10880.
17. Nair, J.K., Attarwala, H., Sehgal, A., Wang, Q., Aluri, K., Zhang, X., Gao, M., Liu, J., Indrakanti, R., Schofield, S., et al. (2017). Impact of enhanced metabolic stability on pharmacokinetics and pharmacodynamics of GalNAc-siRNA conjugates. Nucleic Acids Res. *45*, 10969–10977.

18. Fitzgerald, K., White, S., Borodovsky, A., Bettencourt, B.R., Strahs, A., Clausen, V., Wijngaard, P., Horton, J.D., Taubel, J., Brooks, A., et al. (2017). A Highly Durable RNAi Therapeutic Inhibitor of PCSK9. N. Engl. J. Med. *376*, 41–51.

19. Hassler, M.R., Turanov, A.A., Alterman, J.F., Haraszti, R.A., Coles, A.H., Osborn, M.F., Echeverria, D., Nikan, M., Salomon, W.E., Roux, L., et al. (2018). Comparison of partially and fully chemically-modified siRNA in conjugate-mediated delivery in vivo. Nucleic Acids Res. *46*, 2185–2196.

20. Osborn, M.F., Coles, A.H., Biscans, A., Haraszti, R.A., Roux, L., Davis, S., Ly, S., Echeverria, D., Hassler, M.R., Godinho, B.M.D.C., et al. (2019). Hydrophobicity drives the systemic distribution of lipid-conjugated siRNAs via lipid transport pathways. Nucleic Acids Res. *47*, 1070–1081.

21. Flierl, U., Nero, T.L., Lim, B., Arthur, J.F., Yao, Y., Jung, S.M., Gitz, E., Pollitt, A.Y., Zaldivia, M.T.K.K., Jandrot-Perrus, M., et al. (2015). Phosphorothioate backbone modifications of nucleotide-based drugs are potent platelet activators. J. Exp. Med. *212*, 129–137.

22. Echevarría, L., Aupy, P., Relizani, K., Bestetti, T., Griffith, G., Blandel, F., Komisarski, M., Haeberli, A., Svinartchouk, F., Garcia, L., and Goyenvalle, A. (2019). Evaluating the Impact of Variable Phosphorothioate Content in Tricyclo-DNA Antisense Oligonucleotides in a Duchenne Muscular Dystrophy Mouse Model. Nucleic Acid Ther. *29*, 148–160.

23. Migawa, M.T., Shen, W., Wan, W.B., Vasquez, G., Oestergaard, M.E., Low, A., De Hoyos, C.L., Gupta, R., Murray, S., Tanowitz, M., et al. (2019). Site-specific replacement of phosphorothioate with alkyl phosphonate linkages enhances the therapeutic profile of gapmer ASOs by modulating interactions with cellular proteins. Nucleic Acids Res. *47*, 5465–5479.

24. Stalder, L., Heusermann, W., Sokol, L., Trojer, D., Wirz, J., Hean, J., Fritzsche, A., Aeschimann, F., Pfanzagl, V., Basselet, P., et al. (2013). The rough endoplasmatic reticulum is a central nucleation site of siRNA-mediated RNA silencing. EMBO J. *32*, 1115–1127.

25. Alterman, J.F., Hall, L.M., Coles, A.H., Hassler, M.R., Didiot, M.-C., Chase, K., Abraham, J., Sottosanti, E., Johnson, E., Sapp, E., et al. (2015). Hydrophobically Modified siRNAs Silence Huntingtin mRNA in Primary Neurons and Mouse Brain. Mol. Ther. Nucleic Acids *4*, e266.

26. Nykänen, A., Haley, B., and Zamore, P.D. (2001). ATP requirements and small interfering RNA structure in the RNA interference pathway. Cell *107*, 309–321.

27. Khvorova, A., Reynolds, A., and Jayasena, S.D. (2003). Functional siRNAs and miRNAs exhibit strand bias. Cell *115*, 209–216.

28. Chang, C.I., Yoo, J.W., Hong, S.W., Lee, S.E., Kang, H.S., Sun, X., Rogoff, H.A., Ban, C., Kim, S., Li, C.J., and Lee, D.K. (2009). Asymmetric shorter-duplex siRNA structures trigger efficient gene silencing with reduced nonspecific effects. Mol. Ther. *17*, 725–732.

29. Behlke, M.A. (2008). Chemical modification of siRNAs for in vivo use. Oligonucleotides *18*, 305–319.

30. Alagia, A., Jorge, A.F., Aviñó, A., Cova, T.F.G.G., Crehuet, R., Grijalvo, S., Pais, A.A.C.C., and Eritja, R. (2018). Exploring PAZ/3′-overhang interaction to improve siRNA specificity. A combined experimental and modeling study. Chem. Sci. (Camb.) *9*, 2074–2086.

31. Sheu-Gruttadauria, J., and MacRae, I.J. (2017). Structural Foundations of RNA Silencing by Argonaute. J. Mol. Biol. *429*, 2619–2639.

32. Schirle, N.T., and MacRae, I.J. (2012). The crystal structure of human Argonaute2. Science *336*, 1037–1040.

33. Schirle, N.T., Sheu-Gruttadauria, J., and MacRae, I.J. (2014). Structural basis for microRNA targeting. Science *346*, 608–613.

34. Geary, R.S., Norris, D., Yu, R., and Bennett, C.F. (2015). Pharmacokinetics, biodistribution and cell uptake of antisense oligonucleotides. Adv. Drug Deliv. Rev. *87*, 46–51.

35. Neidle, S. (2008). Principles of Nucleic Acid Structure (Elsevier).

36. Crooke, S.T., Wang, S., Vickers, T.A., Shen, W., and Liang, X.H. (2017). Cellular uptake and trafficking of antisense oligonucleotides. Nat. Biotechnol. *35*, 230–237.

37. Turanov, A.A., Lo, A., Hassler, M.R., Makris, A., Ashar-Patel, A., Alterman, J.F., Coles, A.H., Haraszti, R.A., Roux, L., Godinho, B.M.D.C., et al. (2018). RNAi modulation of placental sFLT1 for the treatment of preeclampsia. Nat. Biotechnol. *36*, 1164–1173.

38. Chernikov, I.V., Vlassov, V.V., and Chernolovskaya, E.L. (2019). Current Development of siRNA Bioconjugates: From Research to the Clinic. Front. Pharmacol. *10*, 444.

39. Sahay, G., Querbes, W., Alabi, C., Eltoukhy, A., Sarkar, S., Zurenko, C., Karagiannis, E., Love, K., Chen, D., Zoncu, R., et al. (2013). Efficiency of siRNA delivery by lipid nanoparticles is limited by endocytic recycling. Nat. Biotechnol. *31*, 653–658.

40. Gilleron, J., Querbes, W., Zeigerer, A., Borodovsky, A., Marsico, G., Schubert, U., Manygoats, K., Seifert, S., Andree, C., Stöter, M., et al. (2013). Image-based analysis of lipid nanoparticle-mediated siRNA delivery, intracellular trafficking and endosomal escape. Nat. Biotechnol. *31*, 638–646.

41. Wittrup, A., Ai, A., Liu, X., Hamar, P., Trifonova, R., Charisse, K., Manoharan, M., Kirchhausen, T., and Lieberman, J. (2015). Visualizing lipid-formulated siRNA release from endosomes and target gene knockdown. Nat. Biotechnol. *33*, 870–876.

42. Kilchrist, K.V., Dimobi, S.C., Jackson, M.A., Evans, B.C., Werfel, T.A., Dailing, E.A., Bedingfield, S.K., Kelly, I.B., and Duvall, C.L. (2019). Gal8 Visualization of Endosome Disruption Predicts Carrier-Mediated Biologic Drug Intracellular Bioavailability. ACS Nano *13*, 1136–1152.

43. Prakash, T.P., Mullick, A.E., Lee, R.G., Yu, J., Yeh, S.T., Low, A., Chappell, A.E., Østergaard, M.E., Murray, S., Gaus, H.J., et al. (2019). Fatty acid conjugation enhances potency of antisense oligonucleotides in muscle. Nucleic Acids Res. *47*, 6029–6044.

44. Biscans, A., Coles, A., Echeverria, D., and Khvorova, A. (2019). The valency of fatty acid conjugates impacts siRNA pharmacokinetics, distribution, and efficacy in vivo. J. Control. Release *302*, 116–125.

45. Juliano, R.L. (2018). Intracellular Trafficking and Endosomal Release of Oligonucleotides: What We Know and What We Don't. Nucleic Acid Ther. *28*, 166–177.

46. Geary, R.S., Wancewicz, E., Matson, J., Pearce, M., Siwkowski, A., Swayze, E., and Bennett, F. (2009). Effect of dose and plasma concentration on liver uptake and pharmacologic activity of a 2′-methoxyethyl modified chimeric antisense oligonucleotide targeting PTEN. Biochem. Pharmacol. *78*, 284–291.

47. Buntz, A., Killian, T., Schmid, D., Seul, H., Brinkmann, U., Ravn, J., Lindholm, M., Knoetgen, H., Haucke, V., and Mundigl, O. (2019). Quantitative fluorescence imaging determines the absolute number of locked nucleic acid oligonucleotides needed for suppression of target gene expression. Nucleic Acids Res. *47*, 953–969.

48. Wagenaar, T.R., Tolstykh, T., Shi, C., Jiang, L., Zhang, J., Li, Z., Yu, Q., Qu, H., Sun, F., Cao, H., et al. (2015). Identification of the endosomal sorting complex required for transport-I (ESCRT-I) as an important modulator of anti-miR uptake by cancer cells. Nucleic Acids Res. *43*, 1204–1215.

49. Dowdy, S.F. (2017). Overcoming cellular barriers for RNA therapeutics. Nat. Biotechnol. *35*, 222–229.

50. Morrissey, D.V., Lockridge, J.A., Shaw, L., Blanchard, K., Jensen, K., Breen, W., Hartsough, K., Machemer, L., Radka, S., Jadhav, V., et al. (2005). Potent and persistent in vivo anti-HBV activity of chemically modified siRNAs. Nat. Biotechnol. *23*, 1002–1007.

51. Song, J.J., Liu, J., Tolia, N.H., Schneiderman, J., Smith, S.K., Martienssen, R.A., Hannon, G.J., and Joshua-Tor, L. (2003). The crystal structure of the Argonaute2 PAZ domain reveals an RNA binding motif in RNAi effector complexes. Nat. Struct. Biol. *10*, 1026–1032.

52. Ma, J.-B.B., Ye, K., and Patel, D.J. (2004). Structural basis for overhang-specific small interfering RNA recognition by the PAZ domain. Nature *429*, 318–322.

53. Frazier, K.S. (2015). Antisense oligonucleotide therapies: the promise and the challenges from a toxicologic pathologist's perspective. Toxicol. Pathol. *43*, 78–89.

54. Chi, X., Gatti, P., and Papoian, T. (2017). Safety of antisense oligonucleotide and siRNA-based therapeutics. Drug Discov. Today *22*, 823–833.

55. Tabrizi, S.J., Leavitt, B.R., Landwehrmeyer, G.B., Wild, E.J., Saft, C., Barker, R.A., Blair, N.F., Craufurd, D., Priller, J., Rickards, H., et al.; Phase 1–2a IONIS-HTTRx Study Site Teams (2019). Targeting Huntingtin Expression in Patients with Huntington's Disease. N. Engl. J. Med. *380*, 2307–2316.

56. Wang, S., Sun, H., Tanowitz, M., Liang, X.H., and Crooke, S.T. (2017). Intra-endosomal trafficking mediated by lysobisphosphatidic acid contributes to intracellular release of phosphorothioate-modified antisense oligonucleotides. Nucleic Acids Res. *45*, 5309–5322.

57. Liang, X.H., Sun, H., Nichols, J.G., Allen, N., Wang, S., Vickers, T.A., Shen, W., Hsu, C.W., and Crooke, S.T. (2018). COPII vesicles can affect the activity of antisense oligonucleotides by facilitating the release of oligonucleotides from endocytic pathways. Nucleic Acids Res. *46*, 10225–10245.

58. Linnane, E., Davey, P., Zhang, P., Puri, S., Edbrooke, M., Chiarparin, E., Revenko, A.S., Macleod, A.R., Norman, J.C., and Ross, S.J. (2019). Differential uptake, kinetics and mechanisms of intracellular trafficking of next-generation antisense oligonucleotides across human cancer cell lines. Nucleic Acids Res. *47*, 4375–4392.

59. Schneider, C.A., Rasband, W.S., and Eliceiri, K.W. (2012). NIH Image to ImageJ: 25 years of image analysis. Nat. Methods *9*, 671–675.

60. Schindelin, J., Arganda-Carreras, I., Frise, E., Kaynig, V., Longair, M., Pietzsch, T., Preibisch, S., Rueden, C., Saalfeld, S., Schmid, B., et al. (2012). Fiji: an open-source platform for biological-image analysis. Nat. Methods *9*, 676–682.

61. Rueden, C.T., Schindelin, J., Hiner, M.C., DeZonia, B.E., Walter, A.E., Arena, E.T., and Eliceiri, K.W. (2017). ImageJ2: ImageJ for the next generation of scientific image data. BMC Bioinformatics *18*, 529.

OMTN, Volume *21*

Supplemental Information

Single-Stranded Phosphorothioated Regions Enhance Cellular Uptake of Cholesterol-Conjugated siRNA but Not Silencing Efficacy

Socheata Ly, Dimas Echeverria, Jacquelyn Sousa, and Anastasia Khvorova

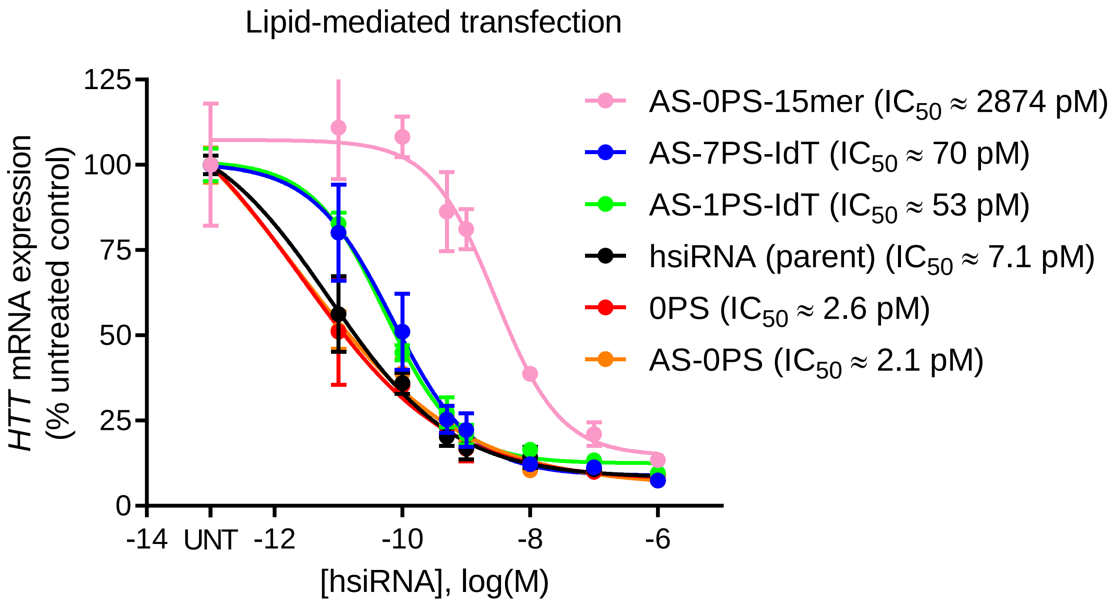

Supplementary Figure 1. PS modifications used in this study are tolerated by RISC, but not a shorter 15-15mer or oligos containing 3′ idT. Seven-point dose response performed in HeLa cells transfected with RNAiMAX. *HTT* mRNA levels were quantified by QuantiGene Assay (Thermo Fisher) after 72 h treatment with hsiRNA. Representative data shown as mean ± SD (n = 3 technical replicates) from at least two independent experiments. UNT = untreated.

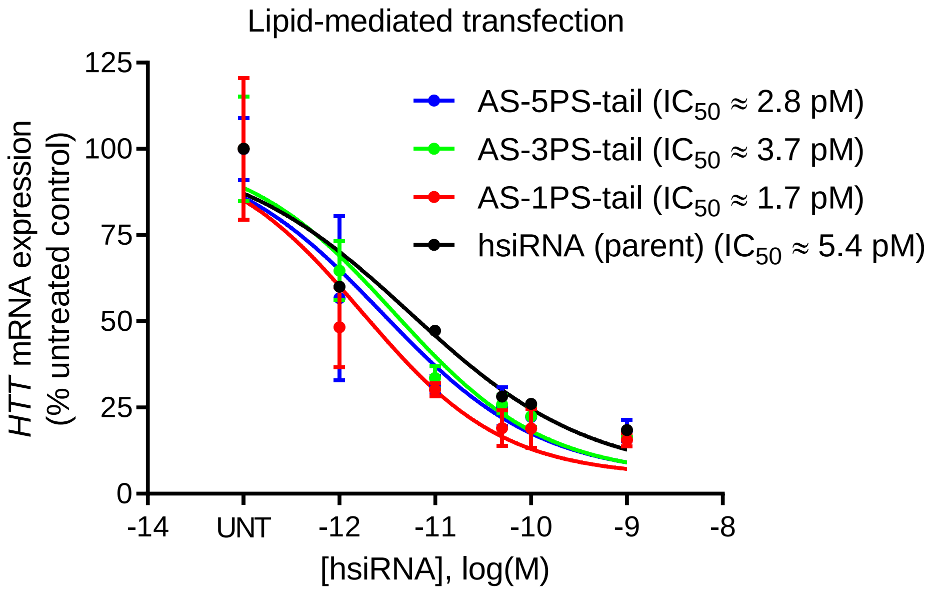

Supplementary Figure 2. Oligos containing reduced PS tails load into RISC as well as the parent compound. Seven-point dose response performed in HeLa cells transfected with RNAiMAX. *HTT* mRNA levels were quantified by QuantiGene Assay (Thermo Fisher) after 72 h treatment with hsiRNA. Representative data shown as mean ± SD (n = 3 technical replicates) from at least two independent experiments. UNT = untreated.

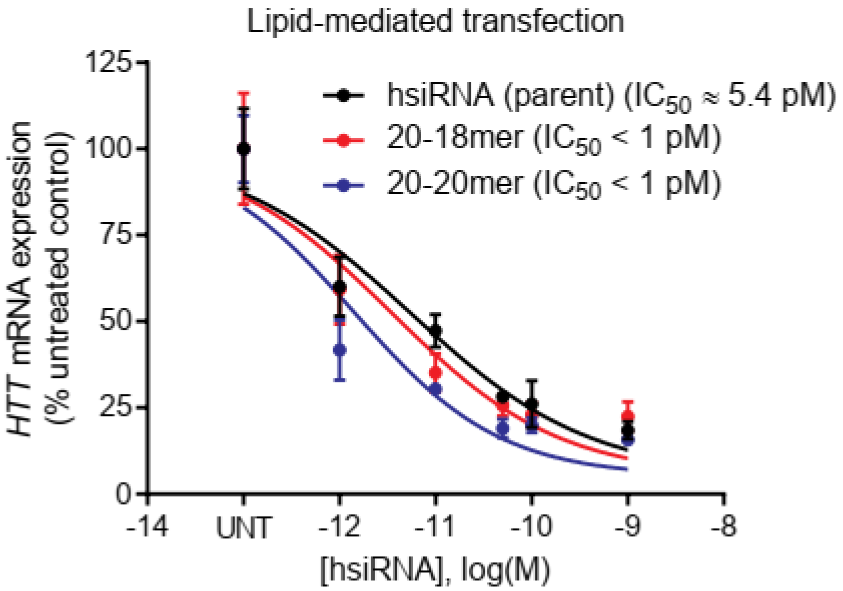

Supplementary Figure 3. 20-20mer and 20-18mer oligos can efficiently silence mRNA via the RNAi pathway. Seven-point dose response performed in HeLa cells transfected with RNAiMAX. *HTT* mRNA levels were quantified by QuantiGene Assay (Thermo Fisher) after 72 h treatment with hsiRNA. Representative data shown as mean ± SD (n = 3 technical replicates) from at least two independent experiments. UNT = untreated.

oligo	strand	sequence (5′ to 3′)
HTT	antisense	P(mU)#(fU)#(mA)(fA)(mU)(fC)(mU)(fC)(mU)(fU)(mU)(fA)(mC)#(fU)#(mG)#(fA)#(mU)#(fA)#(mU)#(fA)
HTT	sense	CyMN3-(fC)#(mA)#(fG)(mU)(fA)(mA)(fA)(mG)(fA)(mG)(fA)(mU)(fU)#(mA)#(fA)-TegChol
sFLT	antisense	P(mU)#(fA)#(mA)(fA)(mU)(fU)(mU)(fG)(mG)(fA)(mG)(fA)(mU)#(fC)#(mC)#(fG)#(mA)#(fG)#(mA)#(fG)
sFLT	sense	CyMN3-(fG)#(mG)#(fA)(mU)(fC)(mU)(fC)(mC)(fA)(mA)(fA)(mU)(fU)#(mU)#(fA)-TegChol
AS-1PS	antisense	P(mU)#(fU)#(mA)(fA)(mU)(fC)(mU)(fC)(mU)(fU)(mU)(fA)(mC)(fU)(mG)(fA)(mU)(fA)(mU)#(fA)
AS-1PS	sense	CyMN3-(fC)#(mA)#(fG)(mU)(fA)(mA)(fA)(mG)(fA)(mG)(fA)(mU)(fU)#(mA)#(fA)-TegChol
AS-3PS	antisense	P(mU)#(fU)#(mA)(fA)(mU)(fC)(mU)(fC)(mU)(fU)(mU)(fA)(mC)(fU)(mG)(fA)(mU)#(fA)#(mU)#(fA)
AS-3PS	sense	CyMN3-(fC)#(mA)#(fG)(mU)(fA)(mA)(fA)(mG)(fA)(mG)(fA)(mU)(fU)#(mA)#(fA)-TegChol
AS-5PS	antisense	P(mU)#(fU)#(mA)(fA)(mU)(fC)(mU)(fC)(mU)(fU)(mU)(fA)(mC)(fU)(mG)#(fA)#(mU)#(fA)#(mU)#(fA)
AS-5PS	sense	CyMN3-(fC)#(mA)#(fG)(mU)(fA)(mA)(fA)(mG)(fA)(mG)(fA)(mU)(fU)#(mA)#(fA)-TegChol
20-20mer	antisense	P(mU)#(fA)#(mA)(fA)(mU)(fU)(mU)(fG)(mG)(fA)(mG)(fA)(mU)#(fC)#(mC)#(fG)#(mA)#(fG)#(mA)#(fG)
20-20mer	sense	CyMN3-(mU)#(fA)#(mU)(fA)(mU)(fC)(mA)(fG)(mU)(fA)(mA)(fA)(mG)(fA)(mG)(fA)(mU)(fU)#(mA)#(fA)-TegChol
20-18mer	antisense	P(mU)#(fA)#(mA)(fA)(mU)(fU)(mU)(fG)(mG)(fA)(mG)(fA)(mU)#(fC)#(mC)#(fG)#(mA)#(fG)#(mA)#(fG)
20-18mer	sense	CyMN3-(mU)#(fA)#(mU)(fC)(mA)(fG)(mU)(fA)(mA)(fA)(mG)(fA)(mG)(fA)(mU)(fU)#(mA)#(fA)-TegChol
HTT15/sFLT5	antisense	P(mU)#(fU)#(mA)(fA)(mU)(fC)(mU)(fC)(mU)(fU)(mU)(fA)(mC)#(fU)#(mG)#(fG)#(mA)#(fG)#(mA)#(fG)
HTT15/sFLT5	sense	CyMN3-(fC)#(mA)#(fG)(mU)(fA)(mA)(fA)(mG)(fA)(mG)(fA)(mU)(fU)#(mA)#(fA)-TegChol
sFLT15/HTT5	antisense	P(mU)#(fA)#(mA)(fA)(mU)(fU)(mU)(fG)(mG)(fA)(mG)(fA)(mU)#(fC)#(mC)#(fA)#(mU)#(fA)#(mU)#(fA)
sFLT15/HTT5	sense	CyMN3-(fG)#(mG)#(fA)(mU)(fC)(mU)(fC)(mC)(fA)(mA)(fA)(mU)(fU)#(mU)#(fA)-TegChol
0PS	antisense	P(mU)(fU)(mA)(fA)(mU)(fC)(mU)(fC)(mU)(fU)(mU)(fA)(mC)(fU)(mG)(fA)(mU)(fA)(mU)(fA)
0PS	sense	CyMN3-(fC)(mA)(fG)(mU)(fA)(mA)(fA)(mG)(fA)(mG)(fA)(mU)(fU)(mA)(fA)-TegChol
AS-0PS	antisense	P(mU)(fU)(mA)(fA)(mU)(fC)(mU)(fC)(mU)(fU)(mU)(fA)(mC)(fU)(mG)(fA)(mU)(fA)(mU)(fA)
AS-0PS	sense	CyMN3-(fC)#(mA)#(fG)(mU)(fA)(mA)(fA)(mG)(fA)(mG)(fA)(mU)(fU)#(mA)#(fA)-TegChol
AS-7PS-idT	antisense	P(mU)#(fU)#(mA)(fA)(mU)(fC)(mU)(fC)(mU)(fU)(mU)(fA)(mC)#(fU)#(mG)#(fA)#(mU)#(fA)#(mU)#(reverse dT)
AS-7PS-idT	sense	CyMN3-(fC)#(mA)#(fG)(mU)(fA)(mA)(fA)(mG)(fA)(mG)(fA)(mU)(fU)#(mA)#(fA)-TegChol
AS-1PS-idT	antisense	P(mU)(fU)(mA)(fA)(mU)(fC)(mU)(fC)(mU)(fU)(mU)(fA)(mC)(fU)(mG)(fA)(mU)(fA)(mU)#(reverse dT)
AS-1PS-idT	sense	CyMN3-(fC)#(mA)#(fG)(mU)(fA)(mA)(fA)(mG)(fA)(mG)(fA)(mU)(fU)#(mA)#(fA)-TegChol
AS-0PS-15mer	antisense	P(mU)(fU)(mA)(fA)(mU)(fC)(mU)(fC)(mU)(fU)(mU)(fA)(mC)(fU)(mG)
AS-0PS-15mer	sense	CyMN3-(fC)#(mA)#(fG)(mU)(fA)(mA)(fA)(mG)(fA)(mG)(fA)(mU)(fU)#(mA)#(fA)-TegChol
No cholesterol	antisense	P(mU)#(fU)#(mA)(fA)(mU)(fC)(mU)(fC)(mU)(fU)(mU)(fA)(mC)#(fU)#(mG)#(fA)#(mU)#(fA)#(mU)#(fA)
No cholesterol	sense	CyMN3-(fC)#(mA)#(fG)(mU)(fA)(mA)(fA)(mG)(fA)(mG)(fA)(mU)(fU)#(mA)#(fA)
**KEY**		
P	5′ phosphate	
mN	2′-O-methyl	
fN	2′-flouro	
#	phosphorothioate	
CyMN3	Cy3	
TegChol	tetra ethylene glycol cholesterol	
reverse dT	3′ to 3′ reverse dT	

Supplementary Table 1. List of oligonucleotides used in this study. See Materials and Methods for information on how the oligonucleotides were synthesized. Key: P, 5′ phosphate; mN, 2′-O-methyl; fN, 2′-flouro; #, phosphorothioate, CyMN3, Cy3; TegChol, tetra ethylene glycol cholesterol; reverse dT, 3′ to 3′ reverse dT.
